# The Obligate Intracellular Bacterial Pathogen Anaplasma phagocytophilum Exploits Host Cell Multivesicular Body Biogenesis for Proliferation and Dissemination

**DOI:** 10.1128/mbio.02961-22

**Published:** 2022-11-21

**Authors:** Curtis B. Read, Mary Clark H. Lind, Travis J. Chiarelli, Jerilyn R. Izac, Haley E. Adcox, Richard T. Marconi, Jason A. Carlyon

**Affiliations:** a Department of Microbiology and Immunology, Virginia Commonwealth University Medical Center, School of Medicine, Richmond, Virginia, USA; Yale University School of Medicine

**Keywords:** multivesicular body, multivesicular endosome, *Anaplasma phagocytophilum*, obligate intracellular bacterium, nutritional virulence, vacuolar pathogen, ESCRT, intralumenal vesicle, exosome, exocytosis, Rab27a, Munc13-4, rickettsia

## Abstract

Anaplasma phagocytophilum is the etiologic agent of the emerging infection, granulocytic anaplasmosis. This obligate intracellular bacterium lives in a host cell-derived vacuole that receives membrane traffic from multiple organelles to fuel its proliferation and from which it must ultimately exit to disseminate infection. Understanding of these essential pathogenic mechanisms has remained poor. Multivesicular bodies (MVBs) are late endosomal compartments that receive biomolecules from other organelles and encapsulate them into intralumenal vesicles (ILVs) using endosomal sorting complexes required for transport (ESCRT) machinery and ESCRT-independent machinery. Association of the ESCRT-independent protein, ALIX, directs MVBs to the plasma membrane where they release ILVs as exosomes. We report that the A. phagocytophilum vacuole (ApV) is acidified and enriched in lysobisphosphatidic acid, a lipid that is abundant in MVBs. ESCRT-0 and ESCRT-III components along with ALIX localize to the ApV membrane. siRNA-mediated inactivation of ESCRT-0 and ALIX together impairs A. phagocytophilum proliferation and infectious progeny production. RNA silencing of ESCRT-III, which regulates ILV scission, pronouncedly reduces ILV formation in ApVs and halts infection by arresting bacterial growth. Rab27a and its effector Munc13-4, which drive MVB trafficking to the plasma membrane and subsequent exosome release, localize to the ApV. Treatment with Nexinhib20, a small molecule inhibitor that specifically targets Rab27a to block MVB exocytosis, abrogates A. phagocytophilum infectious progeny release. Thus, A. phagocytophilum exploits MVB biogenesis and exosome release to benefit each major stage of its intracellular infection cycle: intravacuolar growth, conversion to the infectious form, and exit from the host cell.

## INTRODUCTION

Evolution at the host-microbe interface selected for numerous intracellular bacterial pathogens to remain in host cell-derived vacuoles during infection. While this strategy provides protection from cytosolic immune defenses, it presents 2 major challenges: First, the organisms must obtain nutrients that are not readily accessible. Second, upon completing their replication phase, to exit and invade naive cells they must traverse both the vacuolar and plasma membranes. Because vacuole-adapted bacteria are major causes of disease in terms of incidence and severity for which no vaccines exist and treatment options are limited, defining how they address these challenges to survive, replicate, and disseminate can yield fundamental insights into their molecular pathogenesis and potentially identify new host-directed therapeutic targets.

Anaplasma phagocytophilum is an *Ixodes* spp. tick-transmitted obligate intracellular bacterium that proliferates in membrane-bound inclusions of neutrophils and bone marrow progenitor cells (1, 2). A. phagocytophilum infection in humans, termed human granulocytic anaplasmosis (HGA), is the second-most common tickborne disease in the United States and occurs throughout much of Europe and Asia (3, 4). HGA can also be transmitted perinatally and via blood transfusion (5). Hospitalization is required for 36% of HGA cases and admittance to intensive care for 7% (4, 6). Although doxycycline or rifampicin is usually effective at treating HGA, the risk of fatal disease is greater when antibiotic therapy is delayed (3, 5). Reliable diagnostic assays are lacking, which, when combined with the infection’s nonspecific onset and potential for becoming severe if not treated early, make it a serious health concern (3).

A. phagocytophilum undergoes a biphasic developmental cycle that initiates when its infectious dense-cored (DC) form binds host cell surface receptors to facilitate its endocytic uptake. In the resulting A. phagocytophilum-occupied vacuole (ApV), the DC converts to the noninfectious replicative reticulate cell (RC) morphotype that divides by binary fission. Between 24 and 32 h, replication ceases followed by RC-to-DC transition and release of DCs by an unknown mechanism to initiate the next infection round (7). The ApV is a promicrobial niche that does not fuse with lysosomes but retains endosomal characteristics such as the ability to acquire endocytosed BSA-gold and major histocompatibility (MHC) class II molecules (8–10). The ApV membrane is heavily decorated with monoubiquitinated proteins throughout infection, forms membrane contact sites with the endoplasmic reticulum (ER), and fuses with autophagosomes (11–14). Vesicles including those derived from the ER, *trans*-Golgi network (TGN), and late endosomes/lysosomes that are enriched in cholesterol and sphingolipids traffic into the ApV lumen (7, 14–20). Bacterial uptake of the lipid cargo from these intralumenal vesicles (ILVs) is critical for A. phagocytophilum replication and infectious progeny generation (15–17, 19, 21, 22). Overall, the ApV serves as a platform for a plethora of host cell interactions, including ILV delivery from multiple organelles, that are essential for A. phagocytophilum proliferation, dissemination, and, hence, its success as an endosymbiont and disease agent. How it does so has long remained undefined.

Multivesicular bodies (MVBs), also referred to as multivesicular endosomes, are intermediate compartments in the endosomal system that form when a portion of the limiting membrane of an endosome invaginates and buds into its own lumen (23, 24). ILV generation initiates in the early endosome and continues as the organelle acidifies and matures along the endolysosomal pathway. MVBs are dynamic sorting stations that, in addition to containing endocytosed molecules such as MHC class II, receive membrane traffic from the TGN, ER, and other organelles. MVBs form membrane contacts with the ER and can fuse with autophagosomes (23–26). Tagging transmembrane cargo with monoubiquitin targets them to MVBs for ILV sorting (23–25). Consequently, the MVB cytosolic face is decorated with monoubiquitinated proteins. Cargo partitioning into ILVs and ILV formation involves specific sorting machineries, including endosomal sorting complexes required for transport (ESCRT)-0, -I, -II, and -III. ESCRT-0 binds monoubiquitin conjugated to cargo proteins and recruits ESCRT-I, which, in turn, recruits ESCRT-II that acts as a nucleator for ESCRT-III filaments (25, 26). ESCRT-III filaments can also be nucleated by ALG-2 interacting protein X (ALIX), which is part of the ESCRT-independent pathway. Whereas cargo clustering and membrane budding can occur by either ESCRT-dependent or ESCRT-independent mechanisms, both pathways converge at ESCRT-III, which drives membrane deformation and scission of ILVs into the MVB lumen. Sorting of ILVs into MVBs can direct cargo for lysosomal degradation, or MVBs can fuse with the plasma membrane to release ILVs as exosomes. Exosome biogenesis requires ESCRT-III and ALIX. Moreover, ALIX localization to the MVB limiting membrane specifically routes the organelle for exocytosis (26, 27). Exosomes also contain lysobisphosphatidic acid (LBPA), which is unique to MVBs and other late endocytic compartments. LBPA is on the membranes of ILVs, contributes to ILV formation, and controls the fate of cholesterol and sphingolipids. As such, ILVs and exosomes are enriched in cholesterol and sphingolipids (23, 25). MVBs are present in neutrophils and contribute to their secretory capacity (28).

Based on their extensive similarities, we evaluated the hypothesis that the ApV coopts MVB biogenesis and trafficking. In this study, we demonstrate that the ApV is an acidified organelle enriched in LBPA and decorated by ESCRT and ALIX proteins. siRNA-mediated ESCRT-III knockdown significantly impairs ILV generation in the ApV lumen, A. phagocytophilum proliferation, and infectious progeny release. Rab27a and its effector Munc13-4, both of which are required for MVB positioning at the plasma membrane and subsequent exosome release, localize to the ApV membrane. Treatment with Nexinhib20, a small molecule inhibitor of Rab27a that blocks exocytosis, impairs release of infectious A. phagocytophilum DC organisms. The bacterium’s cooption of MVB biogenesis and exosomal trafficking is central to all phases of its intracellular infection cycle. This study provides insight into the evolution of A. phagocytophilum as an obligate intracellular endosymbiont and identifies new potential host-directed targets for therapeutic intervention of granulocytic anaplasmosis.

## RESULTS

### The ApV is enriched in ILVs, LBPA, and is acidified.

**FIG 1:**
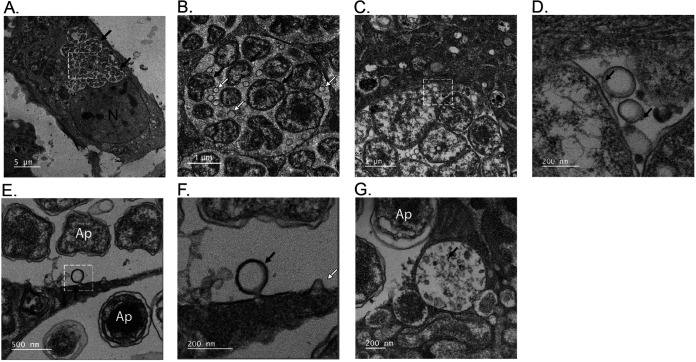
The ApV contains intralumenal vesicles as visualized by TEM. Panels (A), (B), (E), and (F) are transmission electron micrographs of A. phagocytophilum infected RF/6A cells. Panels (C), (D), and (G) are transmission electron micrographs of infected RF/6A cells after incubation with BSA-gold particles for 4 h at 37°C. White hatched boxes in the images in (A), (C), and (E) are enlarged in (B), (D), and (F), respectively. Black arrows in (A) delineate individual ApVs. Black arrows in (B) denote individual bacteria. White arrows in (B) indicate ILVs. Black arrows in (D) point to BSA-gold particles associated with ILVs in close apposition to A. phagocytophilum organisms within an ApV. (E) and (F) A newly formed ILV undergoing scission from the ApV membrane (black arrow in [F]) and a nascent invagination of the ApV membrane (white arrow in [F]) are presented. (G) An MVB containing endocytosed BSA-gold (black arrow) is observed adjacent to an ApV. Scale bars are presented on individual images. Electron micrographs shown are representative of three independent experiments.

To validate if the ApV exhibits MVB characteristics in addition to those already noted (8–19), RF/6A monkey endothelial cells were infected with A. phagocytophilum and examined by transmission electron microscopy (TEM). RF/6A cells are an established model for studying A. phagocytophilum-mammalian host cell interactions as infection-associated phenomena in them are comparable to those in myeloid cells and their large, flat morphology make them ideal for microscopically imaging the ApV (8, 11, 12, 14–18, 29–36). TEM imaging revealed that ApVs harbored numerous ILVs, often in close apposition to A. phagocytophilum organisms (Fig. 1A to D). Many of these ILVs labeled with endocytosed bovine serum albumin (BSA) conjugated to 6-nm colloidal gold particles. Single-membraned ILVs were also observed invaginating from the ApV limiting membrane into the lumen (Fig. 1E and F). Organelles with diameters of approximately 600 nm that were full of ILVs and contained BSA-gold, characteristics of which are consistent with MVBs (24), were also detected proximal to ApVs (Fig. 1G).

**FIG 2:**
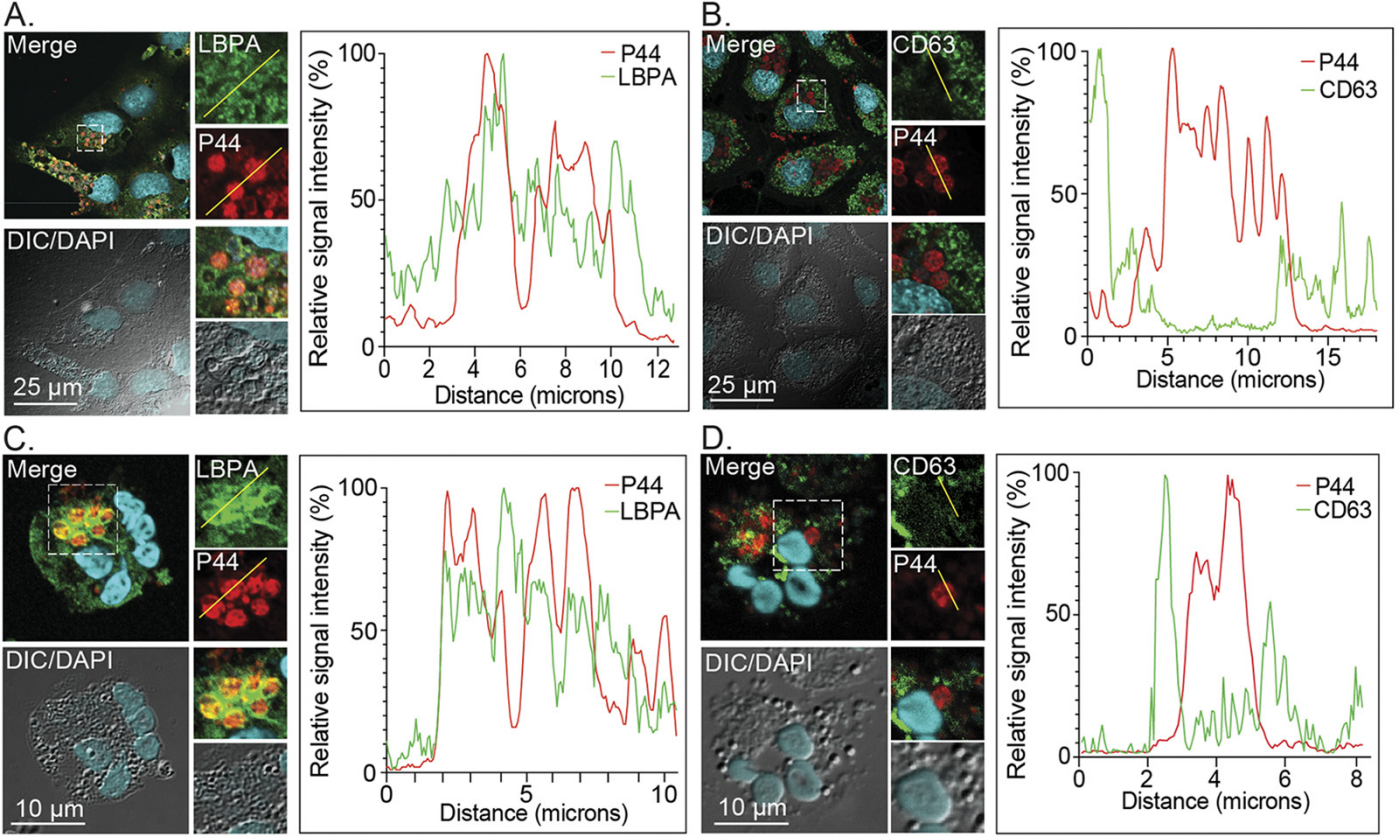
The ApV is enriched in LBPA and lacks CD63. RF/6A cells (A) and (B) and human neutrophils (C) and (D) were infected with A. phagocytophilum for 24 h or 20 h, respectively, after which they were fixed and immunolabeled with antibodies against LBPA (A) and (C), CD63 (B) and (D), and A. phagocytophilum P44 (A) to (D). The cells were stained with DAPI and visualized using LSCM and differential interference contrast microscopy (DIC). The graphs by each panel of micrographs represent the relative signal intensity profiles of green and red pixels along the yellow line (moving left to right) normalized to the highest fluorescence intensity per channel. Scale bars, 25 μm. Results shown are representative of two to four experiments with similar results.

To further characterize the similarities between the ApV and MVBs, A. phagocytophilum infected RF/6A cells and human neutrophils were immunolabeled with antibodies targeting LBPA and CD63, the latter of which is a tetraspanin also known as LAMP-3 (lysosomal-associated membrane protein-3) that is present in internal and limiting membranes of late-stage MVBs destined for lysosomal fusion as well as in lysosomes themselves (37). Antiserum specific for the A. phagocytophilum major surface protein, P44, was used to denote intravacuolar bacteria. The cells were imaged using laser scanning confocal microscopy (LSCM) followed by post-data-acquisition image profiling in which a line was drawn through the confocal micrographs, and the pixel intensities for each immunosignal were graphed for all points along the line. LBPA immunosignal colocalized with P44 immunosignal in both infected cell types (Fig. 2A and C). Consistent with an earlier report (9), CD63 immunosignal did not (Fig. 2B and D). Rab39a, a GTPase that associates with CD63-positive MVBs, is actively recruited by Chlamydia trachomatis to deliver LBPA and sphingomyelin (SM) from MVBs to promote chlamydial intracellular growth (38–40). Reduction of Rab39a cellular levels by treatment with small interfering RNA (siRNA) had no deleterious effect on the proliferation of A. phagocytophilum (Fig. S1), which reinforces that it does not interact with CD63-positive MVBs and indicates that its interactions with the MVB pathway are distinct from C. trachomatis.

10.1128/mbio.02961-22.1 FIG S1 Rab39a is dispensable for A. phagocytophilum infection. RF/6A cells were treated with siRNA targeting Rab39a (siRab39a) or non-targeting siRNA (siNT). (A) At 48 hpi, target knockdown was confirmed by RT-qPCR as the normalized ratio of *rab39a:β-actin* transcript levels from triplicate samples. Data are representative of three experiments with similar results. (B) and (C) Rab39a knockdown and siNT-treated cells were infected with A. phagocytophilum DC organisms and assessed at 48 hpi by Western blotting and densitometry to determine the bacterial load as the mean (±SD) normalized ratios of P44:GAPDH (B) and (C). Statistically significant (**, *P < *0.01) values are indicated. Data are representative of four separate experiments. Download FIG S1, TIF file, 0.7 MB. Copyright © 2022 Read et al. 2022 Read et al. https://creativecommons.org/licenses/by/4.0/ This content is distributed under the terms of the Creative Commons Attribution 4.0 International license.

**FIG 3:**
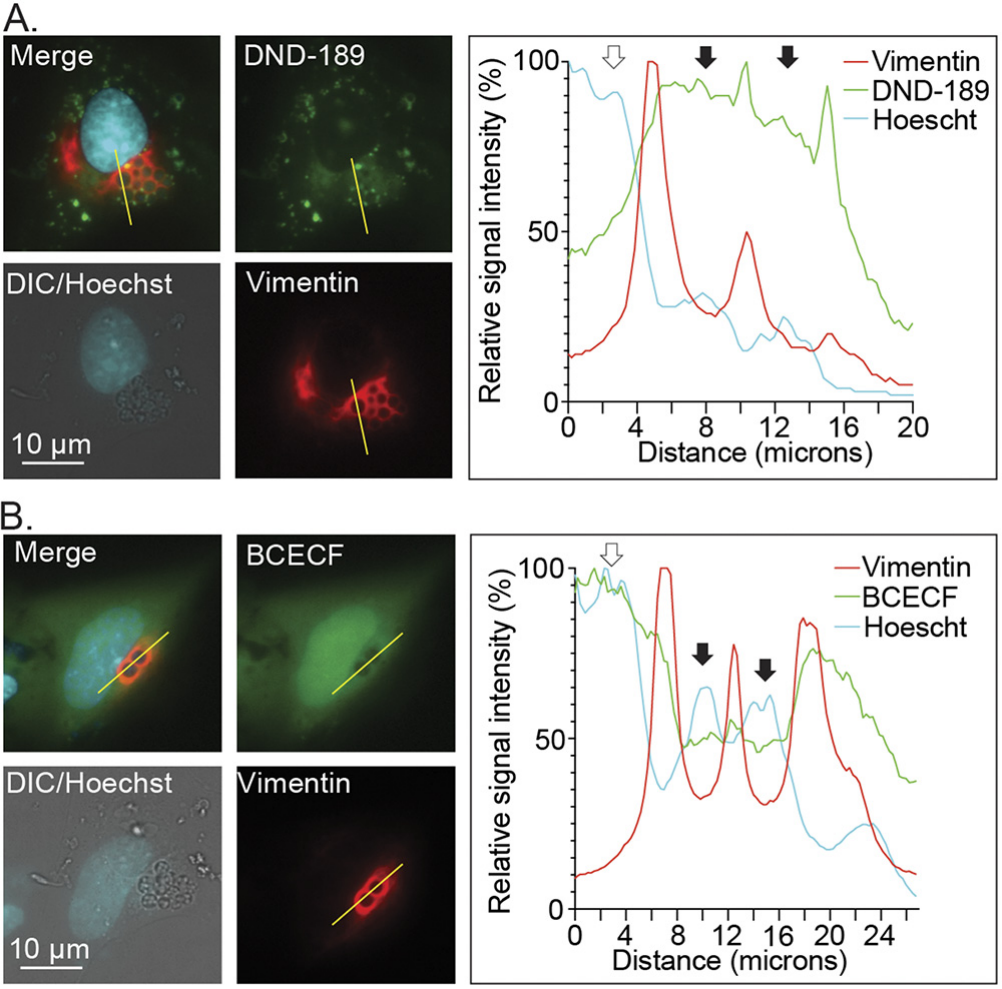
The ApV lumen is acidified. A. phagocytophilum infected RF/6A cells expressing mCherry-vimentin were incubated with LysoSensor Green DND-189 (A) or BCECF (B), stained with Hoechst, and imaged using live cell fluorescence and DIC microscopy. The graphs next to each set of micrographs present the relative signal intensity profiles of red, green, and cyan pixels along the yellow line (moving left to right) normalized to the highest fluorescence intensity per channel. White arrows denote host cell nuclei. Black arrows indicate individual ApVs. Scale bars, 10 μm. Results are representative of at least three independent experiments with similar results.

MVBs have a pH of less than 6.0 (23, 24). The ApV evades fusion with lysosomes and does not accumulate acridine orange, which suggest that its luminal pH exceeds 4.5 to 5.0 (8–10). Yet, the relative pH of this pathogen-occupied organelle had not been defined. To address this knowledge gap, RF/6A cells were transfected to express mCherry-tagged vimentin, an intermediate filament component that wraps the ApV throughout infection (34). The cells were infected with A. phagocytophilum DC organisms. At 24 h, the cells were incubated with LysoSensor Green DND-189, which has a pKa of 5.20, or BCECF (2′,7′-bis-[2-carboxyethyl]-5-[and-6]-carboxyfluorescein, acetoxymethyl ester), which has a pKa of 6.98, and stained with Hoechst. Live cell imaging and post-data-acquisition image profiling revealed DND-189 fluorescence signal colocalization with Hoechst-stained A. phagocytophilum nucleoids bordered by mCherry-vimentin fluorescence signal (Fig. 3A). Consistent with its pKa, the DND-189 signal was weak in the nucleus, which has a neutral pH. In contrast, BCECF fluorescence signal was weak in ApV lumen but strong in the nucleus (Fig. 3B). Overall, these data suggest that the ApV is an organelle that has a moderately acidic pH and that is enriched in ILVs and LBPA, characteristics of which are prototypical for MVBs that are not destined for lysosomal fusion.

### ESCRT-dependent and -independent ILV biogenesis machineries are recruited to the ApV, contribute to A. phagocytophilum replication, and are critical for infectious progeny release.

**FIG 4:**
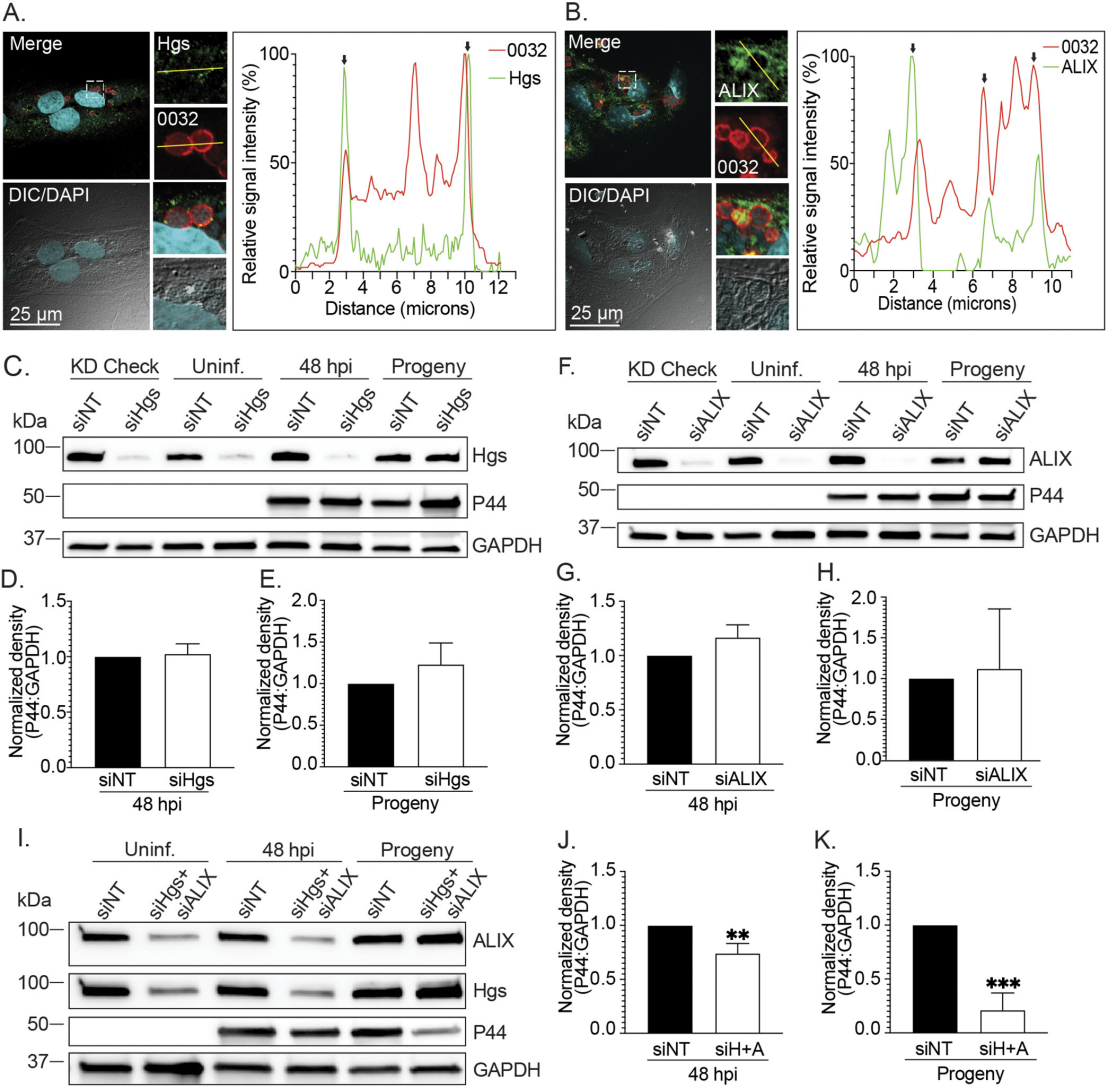
Hgs and ALIX are recruited to the ApV and benefit A. phagocytophilum replication and infectious progeny production. (A) and (B) Hgs and ALIX colocalize with the ApV membrane-localized effector, APH0032. RF/6A cells were infected with A. phagocytophilum for 24 h, after which they were fixed and immunolabeled with antibodies against Hgs (A), ALIX (B), and A. phagocytophilum APH0032 (0032) (A) and (B). The cells were stained with DAPI and examined using LSCM and DIC microscopy. The graphs by each set of micrographs represent the relative signal intensity profiles of green and red pixels along the yellow line (moving left to right) normalized to the highest fluorescence intensity per channel. Black arrows demarcate the ApV membrane. Scale bars, 25 μm. (C) to (K) Knockdown of both Hgs and ALIX hinders A. phagocytophilum proliferation and infectious progeny generation. RF/6A cells were treated with siRNA targeting Hgs (siHgs) (C) to (E), ALIX (siALIX) (F) to (H), both Hgs and ALIX (siH+A) (I) to (K), or non-targeting siRNA (siNT) (C) to (K). At 48 h, one set of each condition was collected to confirm knockdown of the protein of interest (KD check). The other two sets were either mock infected (Uninf.) or incubated with A. phagocytophilum DC organisms for 48 h (48 hpi). All samples were examined by Western blotting using antibodies specific for Hgs, ALIX, P44, and GAPDH. The A. phagocytophilum load was quantified as the mean (±SD) normalized ratio of P44:GAPDH densitometric signals from triplicate samples per condition. To assess for infectious progeny production and release, media from siNT- or target-specific siRNA-treated cells that had been infected with A. phagocytophilum was collected at 48 h postinfection and added to naive RF/6A cells that had not been treated with siRNA. This time point corresponds to when infectious DC bacteria would be present in the media if the bacterial developmental cycle had proceeded normally. Twenty-four h later, recipient cells were assessed for Hgs, ALIX, P44, and GAPDH levels and the A. phagocytophilum load (Progeny). Statistically significant (**, *P < *0.01; ***, *P < *0.001) are indicated. Data shown are representative of three independent experiments.

The importance of the endosomal sorting machineries required for cargo delivery into MVBs to A. phagocytophilum infection was investigated. LSCM analyses of infected RF/6A cells at 24 h determined that both the ESCRT-0 component, Hgs, and the ESCRT-independent protein, ALIX, colocalize with APH0032, an A. phagocytophilum secreted effector that is expressed and localizes to the ApV membrane during late-stage infection, between 20 and 32 h (17, 30) (Fig. 4A and B). The punctate immunolabeling of Hgs on the ApV membrane is consistent with its transient association with the MVB limiting membrane (41). The robust association of ALIX with the ApV membrane and its detection in the ApV lumen agree with its role in complexing and internalizing with ESCRT-III on ILVs as they bud into the MVB lumen. Moreover, ALIX remains associated with and is a marker for ILVs that are destined to be released as exosomes (26, 27, 42). These observations, together with those presented above and previously reported (8–19), confirm that A. phagocytophilum lives within a bacterial-modified MVB in mammalian host cells.

RF/6A cells are amenable to transfection making them favorable for examining the relevance of host proteins to A. phagocytophilum pathobiology (12, 16, 18). To determine the relevance of ESCRT-0 and ALIX to A. phagocytophilum infection, RF/6A cells were treated with Hgs-targeting, ALIX-targeting, or nontargeting siRNA. Hgs and ALIX depletion was confirmed by Western blotting (Fig. 4C and F). Hgs knockdown, ALIX knockdown, and control cells were incubated with A. phagocytophilum DC organisms followed by Western blotting and densitometry analyses 48 h later to quantify the bacterial load as the normalized ratio of P44:GAPDH densitometric signals. Western-blotted lysates were also screened with Hgs and ALIX antibodies to verify that levels of both proteins remained silenced throughout infection. The A. phagocytophilum load was not reduced in either Hgs knockdown or ALIX knockdown cells (Fig. 4C, D, F, and G). To assess for infectious progeny production and release, media was collected from the Hgs siRNA-, ALIX siRNA-, or nontargeting siRNA-treated cells at 48 h, a time point that corresponds to when infectious DC organisms would be present in the media if the A. phagocytophilum infection cycle proceeded normally (7, 17). The media samples were incubated with naive RF/6A cells that had not been treated with siRNA. Examination of the recipient cells 24 h later revealed that RNA silencing of Hgs or ALIX did not inhibit infectious progeny generation and/or release (Fig. 4C, E, F, and H). If A. phagocytophilum benefits from both ESCRT-dependent and -independent cargo delivery into the ApV lumen, then RNA silencing of either pathway alone might be insufficient to impact the bacterium’s fitness as either pathway facilitates ILV formation in MVBs when the other is depleted (24). Indeed, in cells in which both Hgs and ALIX were knocked down, the bacterial load was inhibited by 26% and infectious progeny generation/release was pronouncedly reduced by 79% (Fig. 4I to K). Independent of the aforementioned endosomal sorting machineries, elevated ceramide levels in limiting membranes of MVBs can induce the inward budding that forms ILVs (43). Using GW4869, which blocks this process (43), showed that ceramide dependent ILV biogenesis is dispensable for A. phagocytophilum infection (Fig. S2).

10.1128/mbio.02961-22.2 FIG S2 Pharmacologic inhibition of neutral sphingomyelinase-dependent, ceramide-induced ILV formation does not hinder A. phagocytophilum proliferation. HL-60 cells were infected with A. phagocytophilum in the presence of GW4869 or vehicle (DMSO). At 48 hpi, the bacterial load was assessed by qPCR to determine the bacterial load as the normalized *aph16S*:*β-actin* ratio (A) and by immunofluorescence microscopy to measure the normalized P44 immunosignal mean fluorescence intensity (MFI) (B). Download FIG S2, TIF file, 0.6 MB. Copyright © 2022 Read et al. 2022 Read et al. https://creativecommons.org/licenses/by/4.0/ This content is distributed under the terms of the Creative Commons Attribution 4.0 International license.

### The ESCRT-III component CHMP4 is critical for ILV delivery into the ApV lumen and the A. phagocytophilum infection cycle.

**FIG 5:**
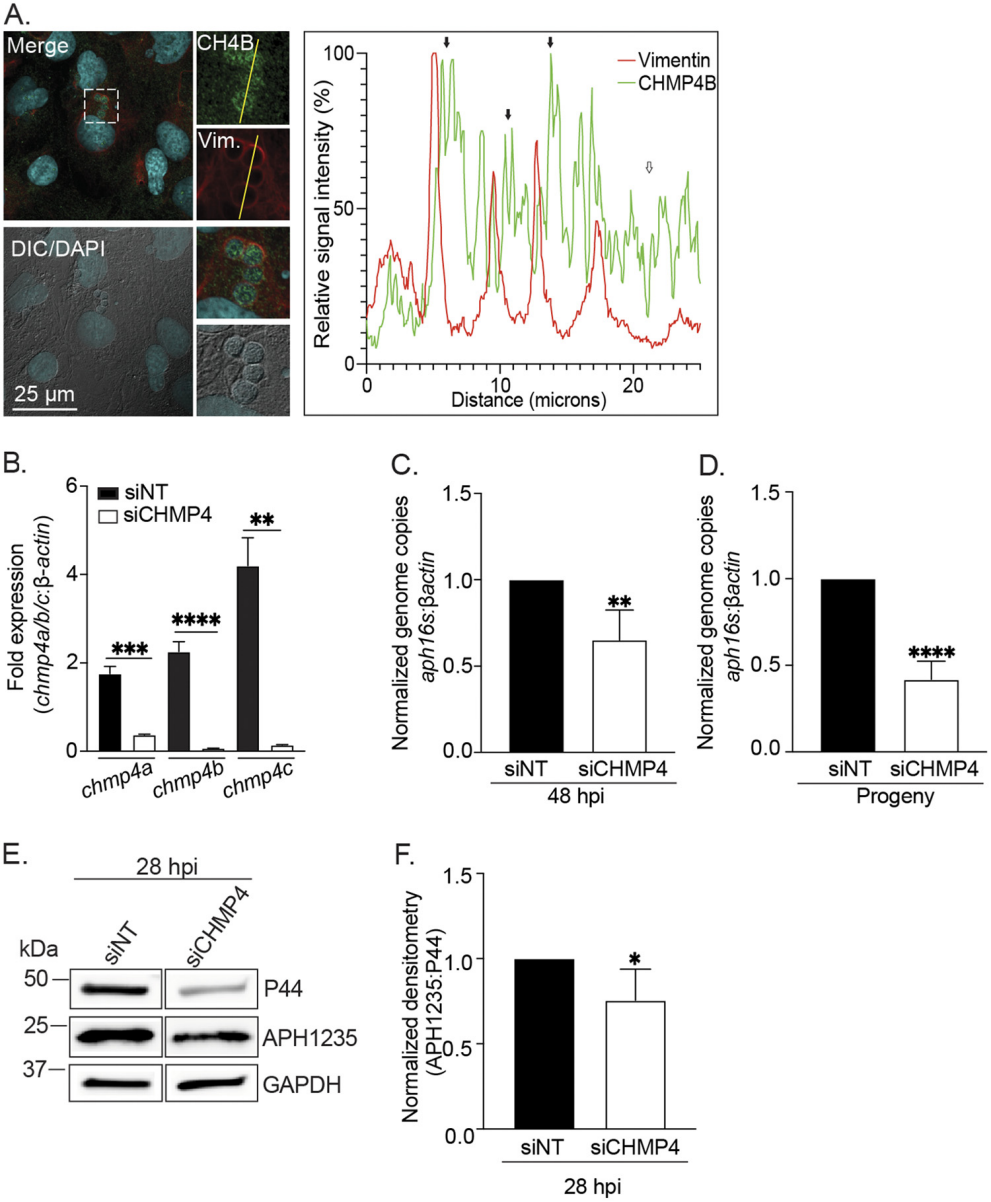
CHMP4 is recruited to the ApV and is critical for infectious progeny release, but not RC-to-DC conversion. (A) CHMP4B localizes to the ApV. RF/6A cells were infected with A. phagocytophilum for 24 h and screened with CHMP4B and vimentin (Vim.) antibodies, stained with DAPI to denote host cell nuclei and A. phagocytophilum nucleoids, and examined using LSCM and DIC microscopy. Scale bar, 25 μm. The graph presents the relative signal intensity profiles of green and red pixels along the yellow line (moving right to left) normalized to the highest fluorescence intensity per channel. (B) and (C) CHMP4 benefits A. phagocytophilum intracellular proliferation. RF/6A cells were treated with an siRNA cocktail targeting CHMP4A, CHMP4B, and CHMP4C (siCHMP4) or siNT. (B) Target knockdown was confirmed by RT-qPCR as the normalized ratios of *chmp4a*, *chmp4b*, and *chmp4c* transcript levels to *β-actin* transcript levels per triplicate samples. (C) siNT-treated and CHMP4 knockdown cells were incubated with A. phagocytophilum DC organisms. At 48 h, the cells were subjected to qPCR to determine the bacterial load as the normalized ratio of the A. phagocytophilum 16S rRNA gene (*aph16S*) to *β-actin*. (D) CHMP4 is important for A. phagocytophilum infectious progeny release. To assess for infectious progeny production and release, media from siNT-treated and CHMP4 knockdown cells that had been infected with A. phagocytophilum was collected at 48 hpi and added to naive RF/6A cells. This time point corresponds to when infectious DC bacteria would be present in the media if the bacterial developmental cycle had proceeded normally. Twenty-four h later, the recipient cells were subjected to qPCR to determine the normalized *aph16S*:*β-actin* ratio. (E) and (F) CHMP4 knockdown does not impair A. phagocytophilum RC-to-DC conversion. siNT-treated and CHMP4 knockdown cells that had been infected with A. phagocytophilum were examined at 28 hpi by Western blotting (E) to determine RC-to-DC conversion as the mean (±SD) normalized ratio of APH1235:P44 densitometric signals (F). Statistically significant (*, *P < *0.05; **, *P < *0.01; ***, *P < *0.001; ****, *P < *0.0001) are indicated. Data presented are representative of 3 to 5 independent experiments.

After ESCRT-dependent or ESCRT-independent machinery has loaded cargo onto the MVB limiting membrane, the ESCRT-III sub-complex drives membrane scission to form ILVs packaged with the cargo into the MVB lumen (26). To determine if CHMP4 assembles on the ApV membrane, A. phagocytophilum infected RF/6A cells were screened with antibodies against vimentin and the ESCRT-III component, charged MVB protein 4B (CHMP4B), and visualized using LSCM. CHMP4B immunosignal was strongest when juxtaposed interior to vimentin immunosignal, an observation that agrees with the ESCRT-III complex being associated with the lumenal face of the ApV limiting membrane (26) (Fig. 5A). To directly examine the relevance of ESCRT-III proteins to A. phagocytophilum infection, we investigated the effects of CHMP4A, CHMP4B, and CHMP4C (CHMP4A/B/C) knockdown on bacterial growth and infectious progeny production. Because available CHMP4 family antibodies were incompatible with Western blotting, reverse transcription-quantitative PCR (RT-qPCR) was used to verify that siRNA treatment achieved near-depletion of *chmp4a*, *chmp4b*, and *chmp4c* mRNA in RF/6A cells (Fig. 5B). At 48 h postinfection (hpi), A. phagocytophilum proliferation in these cells was reduced by 40% and the bacterial load in naive recipient cells after being incubated with media recovered from these cells was reduced by 58% (Fig. 5C and D). APH1235 is an A. phagocytophilum surface protein that contributes to infectivity and is strongly induced near the end of the replication cycle between 28 and 32 h when the bacterium undergoes RC-to-DC conversion (17, 44, 45). APH1235 protein levels in CHMP4A/B/C knockdown cells were reduced by only 25% (Fig. 5E and F).

**FIG 6:**
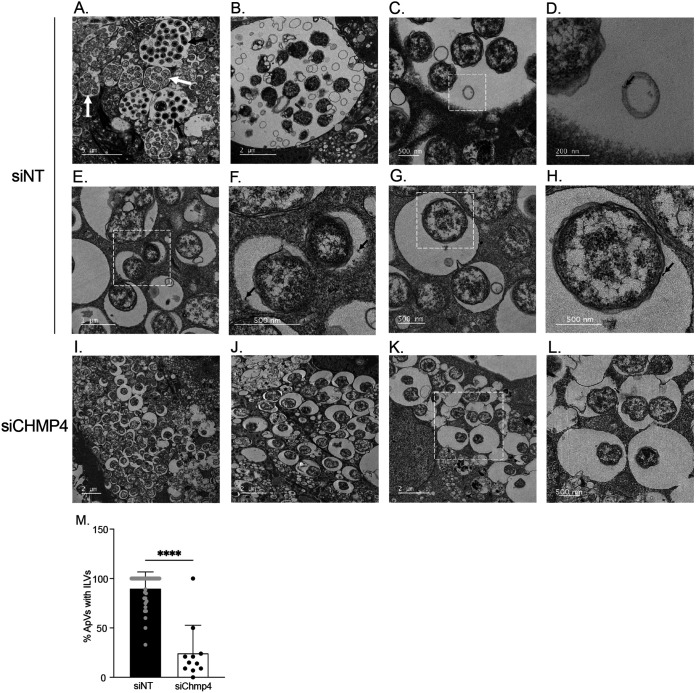
ESCRT-III is essential for ILV delivery into and development of the ApV. siNT-treated RF/6A cells (A) to (H) and CHMP4A/B/C knockdown (siCHMP4) cells (I) to (K) were infected with A. phagocytophilum and examined by TEM at 24 h. Panels (C) to (H) are transmission electron micrographs of infected control cells after incubation with BSA-gold particles for 4 h at 37°C. White hatched boxes in the images in (C), (E), (G), and (K) are enlarged in (D), (F), (H), and (L), respectively. Black arrows and white arrows in (A) denote ApVs harboring DCs or RCs, respectively. Black arrows in (D), (F), and (H) point to BSA-gold particles. Scale bar sizes are indicated. Data are representative of 2 experiments. (M) The percentage of ApVs with ILVs is significantly reduced in siCHMP4 cells. The mean (±SD) percentage of ApVs having ILVs in siCHMP4 and siNT cells was calculated by examining more 200 ApVs in TEM images acquired from grids in different Z-plane sections in two separate experiments. Each gray or black circle corresponds to the percentage of ILV-positive ApVs in individual siNT or siCHMP4 cells. Statistical significance (****, *P < *0.0001) is indicated.

TEM was used to confirm if the impact of CHMP4A/B/C silencing on the A. phagocytophilum infection cycle was associated with inhibition of ILV formation in ApVs. Infected siNT-treated cells contained large ApVs filled with RC or DC bacteria along with smaller ApVs harboring individual or a few RC organisms, which indicates that the infection cycle had proceeded unhindered and that some reinfection events had occurred (Fig. 6A). Also observed in control cells were ApVs containing ILVs (Fig. 6A and B), as well as ApVs with BSA-gold-positive ILVs (Fig. 6C and D), BSA-gold-positive invaginations of the limiting membrane (Fig. 6E and F), and BSA-gold-positive A. phagocytophilum organisms (Fig. 6G and H). In striking contrast, ApVs in CHMP4A/B/C knockdown cells primarily contained single RC bacteria and at most had two (Fig. 6I to K). The percentage of ApVs that contained ILVs in these cells was reduced by 4-fold (Fig. 6I to L). Thus, ESCRT-III is not required for A. phagocytophilum invasion but is crucial for ILV formation in the ApV lumen, which, in turn, is important for bacterial proliferation. Because the A. phagocytophilum infection cycle does not proceed in the absence of ESCRT-III, we conclude that the APH1235 levels that were detected in CHMPH4A/B/C knockdown cells (Fig. 5E and F) were due to its retention by bacteria that had invaded and converted to the RC morphotype but had not replicated.

### Rab27a, Rab27b, and the Rab27a effector Munc13-4 that control MVB exosome formation localize to the ApV.

**FIG 7:**
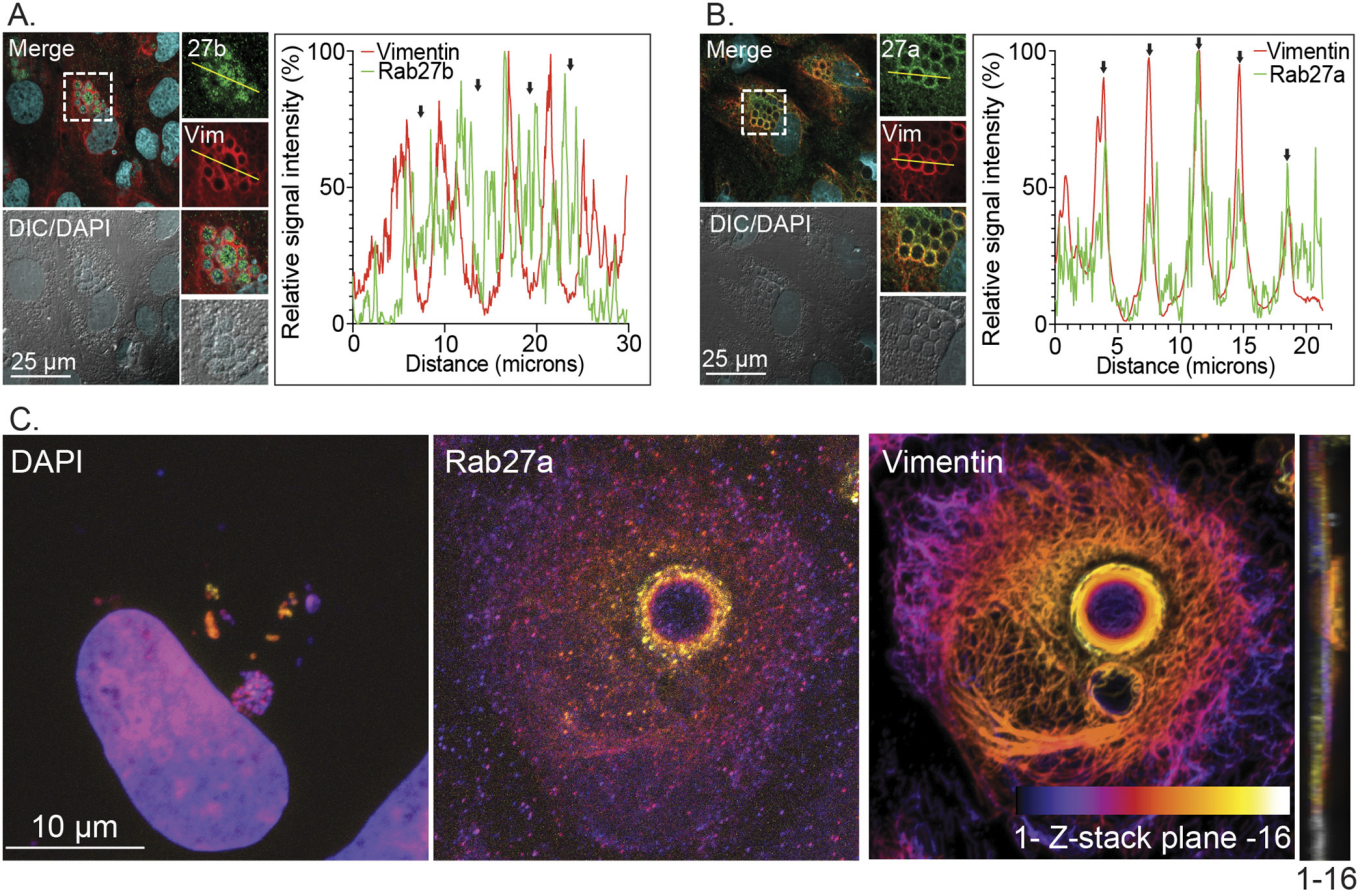
Rab27 isoforms localize to the ApV. At 24 h post-synchronous infection, A. phagocytophilum infected RF/6A cells were immunolabeled with antibodies against Rab27b (A) or Rab27a (B) and (C) and vimentin (A) to (C), stained with DAPI to visualize host cell nuclei and bacterial nucleoids (A) to (C), and examined by LSCM (A) to (C) and DIC microscopy (A) and (B). Scale bar sizes are indicated. The graphs in (A) and (B) represent the relative signal intensity profiles of green and red pixels along the yellow line (moving left to right) normalized to the highest fluorescence intensity per channel. The Z-projection in (C) is an array of the 16 successive Z-plane images from bottom to top that are presented in Fig. S3. A color code, which is indicated at the bottom of the vimentin panel, enabled for assessment of the three-dimensional depth of Rab27a, vimentin, the host cell nucleus, and bacterial nucleoids. For the Z-stack that was used to produce the Z-projection, plane 1 is the bottom-most focal plane while plane 16 is at the top of the stack. An orthogonal view of the Z-stack is provided to the right of the Z-projection. Please note the positions of the Rab27a-positive ApV and its released A. phagocytophilum organisms at the plasma membrane. Data are representative of three experiments with similar results.

The ApV shares traits with MVBs destined to undergo exosome release. It acquires ILVs in an ESCRT-III dependent manner and is ALIX-positive, CD63-negative (9), and non-fusogenic with lysosomes (8–10). To determine if the ApV exhibits additional defining characteristics of pre-exocytic MVBs, we assessed the pathogen-occupied organelle for the association of the small GTPases, Rab27a and Rab27b. Rab27a plays an essential role in exocytosis by associating with and directing MVBs to dock at the plasma membrane as a prerequisite for exosome release (46). Rab27b acts upstream of Rab27a by directing membrane traffic from the TGN to the MVB limiting membrane, which leads to the formation of ILVs carrying TGN cargo in the MVB lumen (47). LSCM analyses detected Rab27b on ApV limiting membranes and within ApVs as a vesicular-like immunolabeling pattern interspersed with DAPI-stained A. phagocytophilum nucleoids (Fig. 7A), a result that is reminiscent of the immunolabeling pattern previously reported for Rab10-positive TGN-derived vesicles within the ApV (17).

10.1128/mbio.02961-22.3 FIG S3 Z-stack series used to produce the Z-projection presented in Fig. 7B. At 24 h post-synchronous infection, A. phagocytophilum infected RF/6A cells were immunolabeled with antibodies against Rab27a and vimentin, stained with DAPI to visualize host cell nuclei and bacterial nucleoids, and examined by LSCM. Sixteen successive images in the X-Y plane along the Z-axis from bottom to top are presented. Download FIG S3, TIF file, 2.7 MB. Copyright © 2022 Read et al. 2022 Read et al. https://creativecommons.org/licenses/by/4.0/ This content is distributed under the terms of the Creative Commons Attribution 4.0 International license.

**FIG 8:**
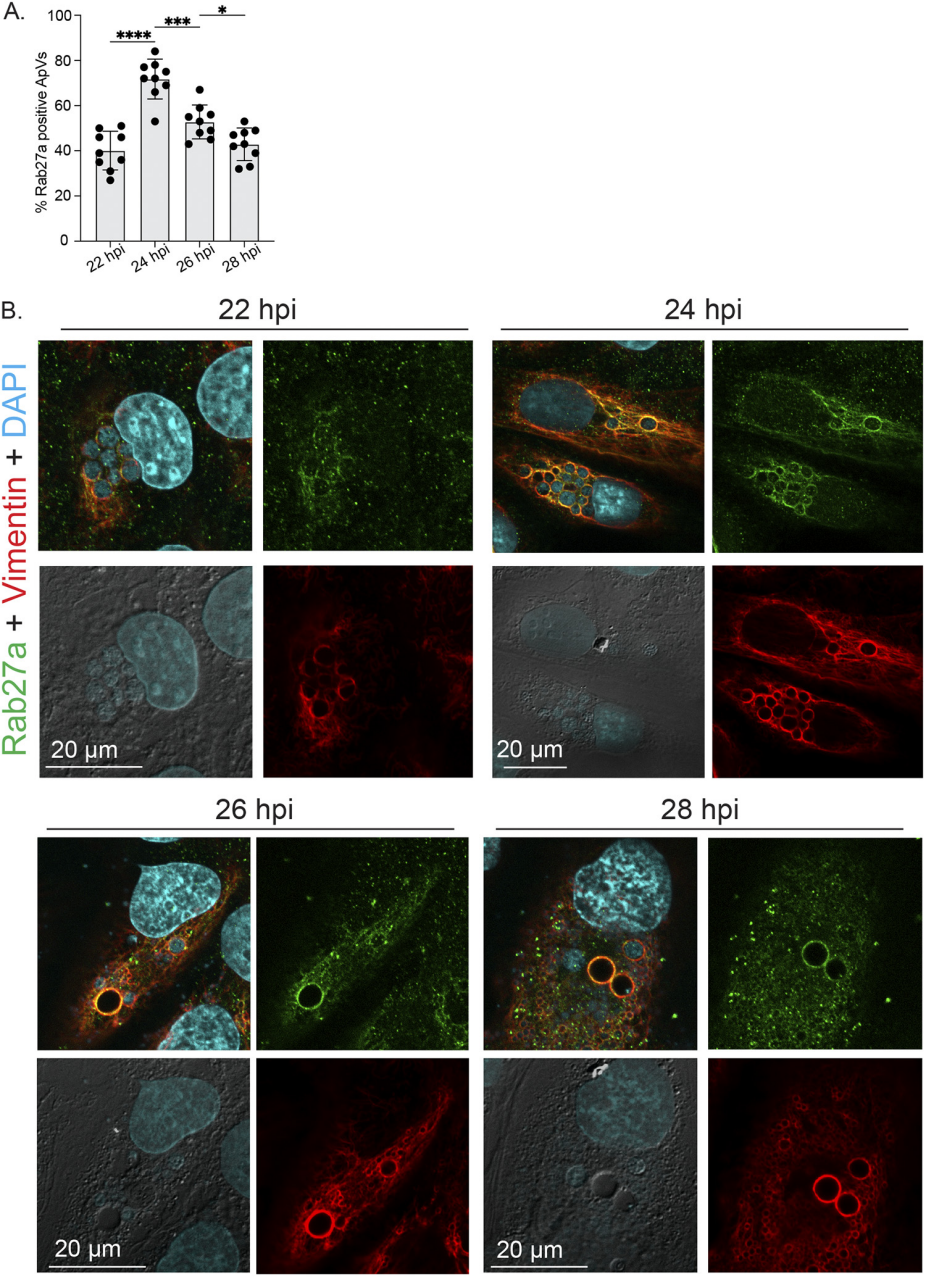
Peak Rab27a localization to ApVs precedes reinfection. RF/6A cells that had been synchronously infected with A. phagocytophilum were immunolabeled with antibodies against Rab27a and vimentin, stained with DAPI to visualize host cell nuclei and bacterial nucleoids, and examined by LSCM and DIC microscopy at 22, 24, 26, and 28 hpi. (A) The mean (±SD) percentage of Rab27a-positive ApVs was calculated from the total number of ApVs from 100 infected cells examined per time point. Each black circle corresponds to the percentage of Rab27a-positive ApVs in individual cells. (B) Representative confocal micrographs and DIC images. Statistically significant (*, *P < *0.05; ***, *P < *0.001; ****, *P < *0.0001) are indicated. Data are representative of three experiments with similar results each performed in triplicate.

The presence of Rab27a on ApVs was also confirmed (Fig. 7B). Z-stack images of infected cells (Fig. S3) were arrayed as Z-projections to which a color code was applied that enabled us to assess three-dimensional depth of Rab27a, vimentin, the host cell nucleus, and bacterial nucleoids. Figure 7C presents a representative cell in which 2 ApVs are present at distinct depths. One is at approximately the same depth as the nucleus, is full of bacteria, and is wrapped in vimentin but has almost no associated Rab27a. The disparity of Rab27a is consistent with this ApV not being positioned at the exocytic zone of the plasma membrane. By contrast, the larger ApV that is positioned at the plasma membrane exhibits robust Rab27a and vimentin localization and comparatively much fewer A. phagocytophilum organisms. Notably, bacteria are around the periphery of this ApV and in the same plane as the plasma membrane. Thus, at the time the cell was fixed and imaged, this Rab27a-positive ApV was positioned at the plasma membrane and releasing its intralumenal A. phagocytophilum organisms to the extracellular milieu in comparable fashion to how an MVB releases exosomes. Next, the experiment was repeated except that Rab27a association with ApVs was monitored over a short time course between 22 and 28 h, a period during which ApVs fully mature and infectious progeny are released (7, 17). The percentage of Rab27a-positive ApVs peaked at 24 h (Fig. 8A). At 26 and 28 h, the largest ApVs in any given cell were Rab27a positive and were mostly devoid of bacteria (Fig. 8B), which is consistent with these vacuoles having released their bacterial cargo. Also, at 28 h, host cells that had large Rab27a positive ApVs also contained numerous ApVs that were comparatively much smaller, vimentin-positive, Rab27a-negative, and harbored few DAPI-positive bacteria. These smaller ApVs would have resulted from multiple reinfection events, each caused by individual bacteria that had been released between 24 and 26 h.

**FIG 9:**
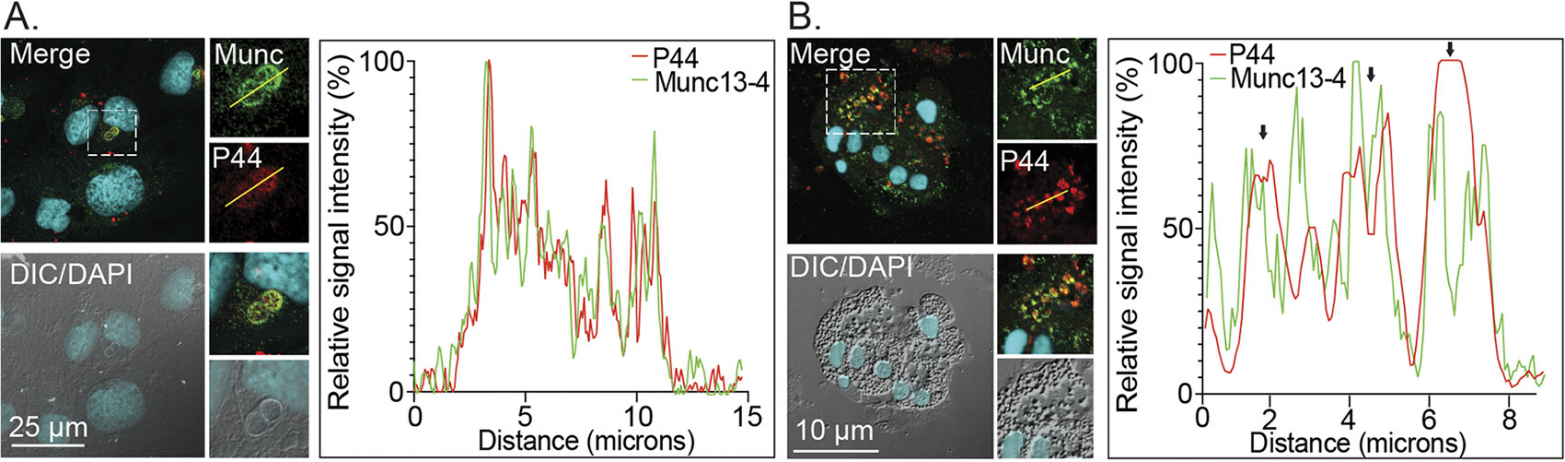
Munc13-4 localizes to the ApV. RF/6A cells and human neutrophils were infected with A. phagocytophilum for 24 h or 20 h, respectively, after which they were fixed, immunolabeled with antibodies against Munc13-4 (Munc) and A. phagocytophilum P44, stained with DAPI, and examined using LSCM and DIC microscopy. Scale bar sizes are indicated. The graphs represent the relative signal intensity profiles of green and red pixels along the yellow line (moving left to right) normalized to the highest fluorescence intensity per channel. Results are representative of 2 to 3 experiments with similar results.

The Rab27a effector Munc13-4 directly controls the fate of exocytosable, Rab27a-positive vesicles in neutrophils and other cell types. It acts as a tethering factor that facilitates docking and subsequent SNARE-mediated fusion of the MVB membrane with the plasma membrane to release ILVs as exosomes (28). Munc13-4 was detected on the ApV membrane proximal to intralumenal A. phagocytophilum organisms in infected RF/6A cells and human neutrophils (Fig. 9).

### The Rab27a small molecule inhibitor, Nexinhib20 inhibits A. phagocytophilum infectious progeny release.

**FIG 10:**
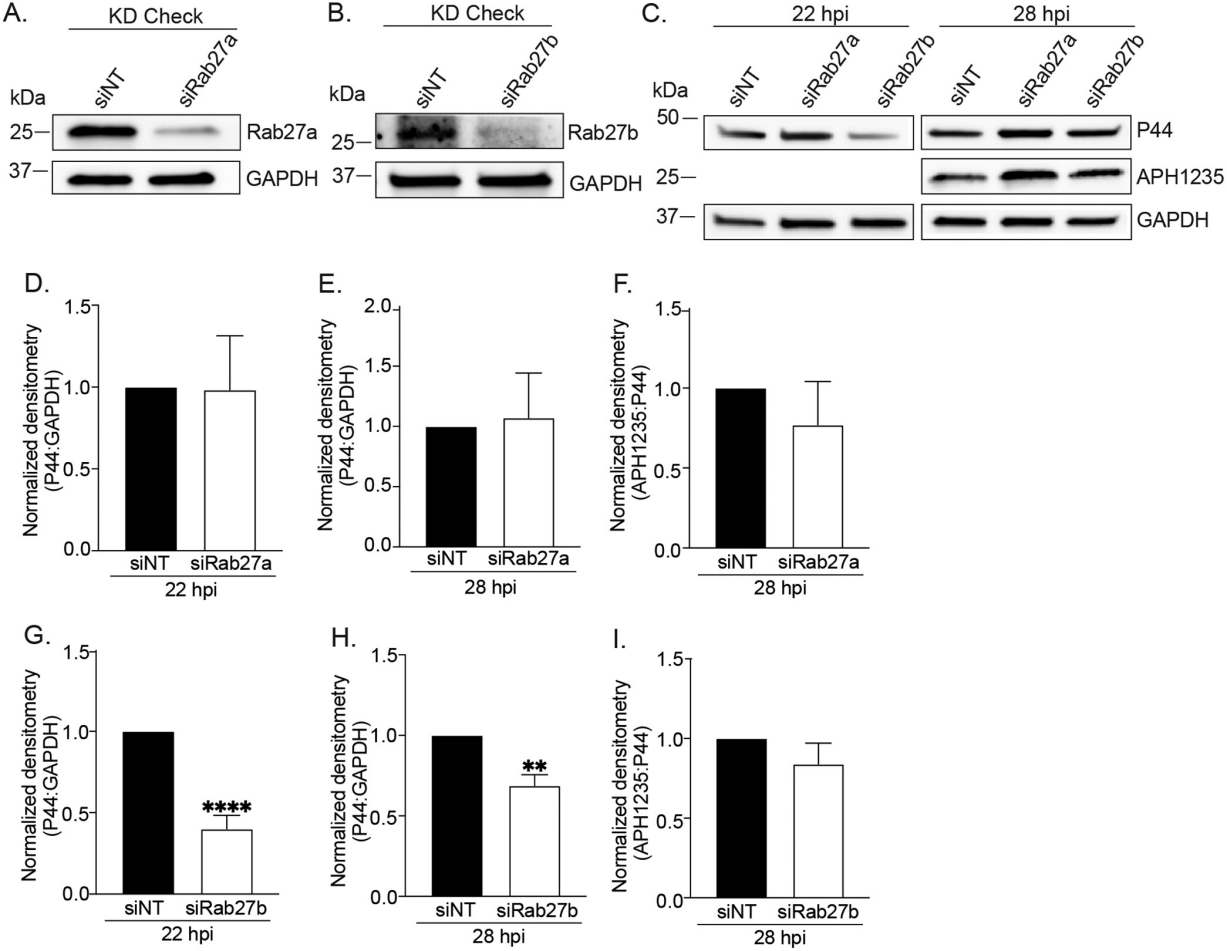
siRNA-mediated knockdown of Rab27b, but not Rab27a inhibits the A. phagocytophilum infection cycle. (A) and (C) to (F) Rab27a knockdown does not impair the A. phagocytophilum infection cycle within the first 28 h. RF/6A cells were treated with Rab27a-specific siRNA (siRab27a) or non-targeting siRNA (siNT). Target knockdown was confirmed at by Western blotting at 48 h (A). Rab27a knockdown and siNT control-treated cells were infected with A. phagocytophilum DC organisms and assessed at the indicated time points by Western blotting (C) and densitometry to determine the bacterial load (D) and (E) and RC-to-DC conversion (F) as the mean (±SD) normalized ratios of P44:GAPDH and APH1235:P44 densitometric signals, respectively. (B), (C), (G) to (I) Rab27b knockdown reduces the A. phagocytophilum load, but not RC-to-DC conversion. RF/6A cells treated with Rab27b-targeting siRNA (siRab27b) or siNT cells were examined by Western blotting for target knockdown at 48 h (B). Rab27b knockdown and control cells were infected with DC organisms and assessed by Western blotting (C) and densitometry to determine the bacterial load (G) and (H) and RC-to-DC conversion (I). Statistically significant (**, *P < *0.01; ****, *P < *0.0001) are indicated. Data presented are representative of 5 separate experiments.

**FIG 11:**
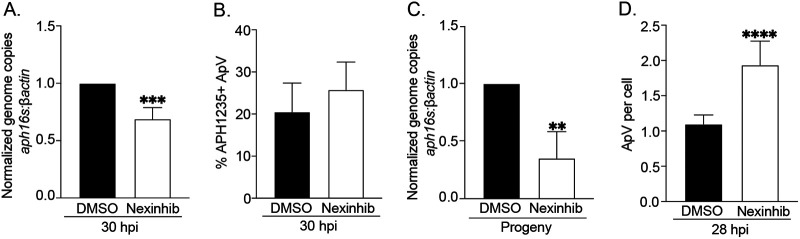
Nexinhib20 inhibits the A. phagocytophilum infection cycle by impeding infectious progeny release. (A) to (C) Nexinhib20 treatment reduces the overall A. phagocytophilum load by inhibiting DC release, but not RC-to-DC conversion. HL-60 cells that had been synchronously infected with A. phagocytophilum were treated with Nexinhib20 or vehicle control (DMSO) starting at 24 h. Six h later, which corresponds to 30 hpi, aliquots were examined by qPCR to determine the bacterial load as the normalized *aph16S*:*β-actin* ratio (A) or immunofluorescence microscopy screening to determine the mean (±SD) percentage of ApVs harboring bacteria that were positive for the DC-specific marker, APH1235 (B). Also at 30 h, media from DMSO- and Nexinhib20-treated cells, which normally would contain naturally liberated infectious DC organisms, was transferred to naive HL-60 cells. Twenty-four h later, the A. phagocytophilum DNA load in the recipient cells was quantified using qPCR (C). (D) Nexinhib20 inhibits ApV exit. HL-60 cells were synchronously infected with A. phagocytophilum and treated with Nexinhib20 or DMSO beginning at 20 h. After 8 h, which corresponds to 28 hpi, the cells were immunolabeled with P44 antibody and the normalized percentage of infected cells and mean (±SD) number of ApVs per cell were determined. Statistically significant (**, *P < *0.01; ***, *P < *0.001; ****, *P < *0.0001) are indicated. Data are representative of three experiments.

To characterize the requirement of Rab27a and Rab27b in A. phagocytophilum infection, both bacterial growth and RC-to-DC differentiation were quantified in RF/6A siRNA knockdown cells. Near-depletion of Rab27a using siRNA did not impede A. phagocytophilum growth in RF/6A cells, but Rab27b knockdown did (Fig. 10A to E, G and H). The latter result is consistent with TGN parasitism benefitting A. phagocytophilum proliferation (17). RC-to-DC differentiation was not affected in either Rab27a or Rab27b knockdown cells (Fig. 10F and I). Because Rab27b can functionally compensate for Rab27a in its absence (48), we reevaluated the relevance of Rab27a to A. phagocytophilum infection in myeloid cells using the Rab27a-specific small molecule inhibitor, neutrophil exocytosis inhibitor 20 (Nexinhib20). This compound binds to Rab27a to prevent it from binding its effector JFC1, which helps position MVBs in the exocytic zone prior to the plasma membrane docking and membrane fusion that the Rab27a-Munc13-4 interaction orchestrates (49). A control experiment verified that Nexinhib20 treatment does not alter HL-60 cell susceptibility to A. phagocytophilum infection (Fig. S4A and B). Next, HL-60 cells were synchronously infected with DC bacteria followed by incubation with Nexinhib20 or vehicle beginning at 24 h. Infection was identical between the groups when treatment was initiated (Fig. S4C and D). Assessment after 6 h revealed that Nexinhib20-treated cells had bacterial DNA loads that were 30% lower than controls (Fig. 11A). Although APH1235 expression was unaltered in Nexinhib20-treated cells, infectivity of media recovered from these cells was reduced by 65% (Fig. 11B and C). Because the time at which the assay was performed corresponded to 30 hpi and DCs begin to exit HL-60 cells around 28 hpi (7), we hypothesized that the higher bacterial load in DMSO-treated cells was due to exocytosis-dependent DC release and reinfection that were, in fact, inhibited in Nexinhib20-treated cells. Accordingly, the experiment was repeated except that Nexinhib20 treatment was initiated at 20 h and the number of ApVs per cell counted at 28 h. Nexinhib20-treated cells had 1.8-fold more ApVs than control cells (Fig. 11D).

10.1128/mbio.02961-22.4 FIG S4 Nexinhib20 control experiments. (A) and (B) Nexinhib20 pretreatment of host cells has no effect on A. phagocytophilum infection. HL-60 cells were pretreated with Nexinhib20 or vehicle (DMSO) for 1 h and incubated with A. phagocytophilum DC organisms in continued presence of the drug. After 1 h, unbound bacteria and treatments were removed by washing in PBS. At 24 hpi, the cells were examined using immunofluorescence microscopy for the normalized percentage of infected cells (A) and mean (±SD) number of ApVs per cell (B). (C) and (D) Confirmation that the bacterial loads were comparable between infected HL-60 cell cultures prior to treatment with DMSO or Nexinhib20 in Fig. 10A and B. Triplicate cultures of A. phagocytophilum infected HL-60 cells that were to be treated with DMSO or Nexinhib20 in the experiment presented in Fig. 10A and B were assessed for the normalized percentage of infected cells (C) and mean (±SD) number of ApVs per cell (D) using immunofluorescence microscopy. (E and F) Assessment of the bacterial load in Nexinhib20- and vehicle-treated cells. As described for Fig. 10 (A and B), HL-60 cells that had been synchronously infected with A. phagocytophilum were treated with Nexinhib20 or DMSO starting at 24 h. Six h later, which corresponds to 30 h post infection (hpi), aliquots of the same samples processed in Fig. 10A and B were immunolabeled with P44 antibody and examined by immunofluorescence microscopy to determine the normalized percentage of infected cells (E) and mean (±SD) number of ApVs per cell (F), respectively. Data are indicative of four experiments each performed in triplicate. Download FIG S4, TIF file, 0.7 MB. Copyright © 2022 Read et al. 2022 Read et al. https://creativecommons.org/licenses/by/4.0/ This content is distributed under the terms of the Creative Commons Attribution 4.0 International license.

Munc13-4 is also a Rab11a effector that regulates trafficking of Rab11-positive recycling endosomes and directs their engagement in exocytosis by inducing tethering at the exocytic active zone (50). Because Rab11a is one of several recycling endosome-associated Rab GTPases present on the ApV membrane (8), its relevance to A. phagocytophilum infection was investigated. Rab11a-depleted cells were uncompromised in the abilities to support A. phagocytophilum infection or yield infectious progeny (Fig. S5). Overall, these data demonstrate that A. phagocytophilum exploits the Rab27a-dependent MVB exosome release pathway to disseminate infection by positioning its vacuole at and fusing with the plasma membrane to release DC organisms to the extracellular milieu.

10.1128/mbio.02961-22.5 FIG S5 Rab11a is dispensable for A. phagocytophilum infection. (A) to (C) Knockdown of Rab11a does not hinder A. phagocytophilum proliferation or infectious progeny release. RF/6A cells were treated with siRNA targeting Rab11a (siRab11a) or non-targeting siRNA (siNT). At 48 h, knockdown was verified by Western blotting (KD check) (A). Duplicate sets of Rab11a knockdown and siNT-treated cells were infected with A. phagocytophilum. At 48 hpi, Western blot analysis was performed using antibodies specific for Rab11a, A. phagocytophilum P44, and β-actin (A). To assess for infectious progeny production and release, media from Rab11a knockdown and control cells was collected at 48 h post infection and added to naïve RF/6A cells that had not been treated with siRNA. This time point corresponds to when infectious DC bacteria would be present in the media if the bacterial developmental cycle had proceeded normally. Twenty-four h later, recipient cells were assessed by Western blotting for Rab11a, β-actin, and P44 (Progeny). (B) and (C) The A. phagocytophilum load in Rab11a knockdown and control cells at 48 hpi (B) and recipient cells incubated with media from infected Rab11 knockdown and control cells (Progeny) (C) was quantified from the Western blot analyses presented in (A) as the mean (±SD) normalized ratio of P44:GAPDH densitometric signals from triplicate samples per condition. (D) and (E) As a complementary approach, Rab11a knockdown and siNT-treated RF/6A cells infected with A. phagocytophilum were screened at 24 hpi by immunofluorescence microscopy using P44 antibody to determine the normalized percentage of infected cells (D) and mean (±SD) number of ApVs per cell. Data are representative of three to four experiments with similar results. Download FIG S5, TIF file, 0.9 MB. Copyright © 2022 Read et al. 2022 Read et al. https://creativecommons.org/licenses/by/4.0/ This content is distributed under the terms of the Creative Commons Attribution 4.0 International license.

## DISCUSSION

A. phagocytophilum exploits MVB biogenesis to benefit each major stage of its intracellular infection cycle: intravacuolar proliferation, RC-to-DC conversion, and infectious progeny release. The ApV is an acidified organelle that accumulates monoubiquitinated cargo (11), ESCRT-dependent sorting machinery, the ESCRT-independent/exosome-restricted protein ALIX, ILVs, LBPA but not CD63 (9), and subsequently Rab27a and Munc13-4. This protein localization pattern and the plasma membrane-trafficking that the ApV undergoes late in infection are identical to how MVBs destined for exosome release diverge from lysosomal fusion and translocate to and fuse with the plasma membrane (26–28, 47, 48, 51). The ApV does not fuse with MVBs as supported by TEM observations showing intact MVBs proximal to but not fusing with ApVs. The ApV does not intercept traffic from MVBs, based on comparisons between it and the C. trachomatis inclusion. C. trachomatis hijacks Rab39a to steal LBPA and SM from MVBs (38–40). Chlamydial proliferation and infectious progeny release do not require ESCRT-I, -II, or -III (52). In contrast, the A. phagocytophilum infection cycle progression is Rab39a-independent, but critically reliant on ESCRT-0, ALIX, and ESCRT-III. These opposing nutritional virulence phenotypes indicate that, unlike C. trachomatis intercepting traffic from MVBs to its inclusion, A. phagocytophilum lives in a modified MVB. A distinguishing characteristic of the ApV from an MVB is its larger size. Indeed, the diameter range of a single A. phagocytophilum bacterium is the same as an MVB (7, 24). Whether the bacterium passively enters the MVB pathway via internalization through known adhesin-receptor interactions (33, 53, 54) or actively does so in a bacterial effector-dependent manner is unknown. Nonetheless, given that the ApV is decorated with bacterial effectors (12, 30, 32, 55–57), is full of ILVs, and traffics like an exocytic MVB, we can conclude that A. phagocytophilum lives in a pathogen-modified and aberrantly large MVB that releases infectious progeny by undergoing exosomal secretion.

This report provides new context for understanding how A. phagocytophilum parasitizes host lipids, namely, cholesterol and sphingolipids. The ApV has long been known to be enriched in cholesterol and the cholesterol transport proteins, Niemann-Pick type C1 (NPC1) and NPC2 (19). It has been proposed that A. phagocytophilum hijacks NPC1, NPC2, and flotillin to its inclusion to acquire cholesterol (16, 19). However, this model needs to be revisited given that all 3 proteins are in MVBs (24, 58). ILVs are enriched in cholesterol and sphingolipids in early stage MVBs but contain higher concentrations of LBPA and ceramide in more mature, acidified MVBs. The decrease in ILV cholesterol content in pre-lysosomal MVBs is due to extraction and transport via NPC1 and NPC2 (24). NPC2 is a soluble protein that binds LBPA on the ILV membrane and extracts cholesterol from ILVs (24, 59). NPC1, which is present on intralumenal vesicles and on the limiting membrane of MVBs, transports cholesterol across the MVB limiting membrane (24, 60). Both proteins are present in the same topological locations in the ApV as in MVBs (16, 19, 24, 60). Thus, A. phagocytophilum may not reroute cholesterol laden NPC1/NPC2 vesicles to its vacuole. Rather, these proteins are naturally present and functioning within MVBs in which the bacterium lives. Moreover, although flotillin interacts with cholesterol and NPC1 (16), it also regulates transfer of ubiquitinated cargo between ESCRT-0 and ESCRT-I (58). The inhibition of A. phagocytophilum proliferation in cells in which flotillin is depleted using siRNA is associated with decreased trafficking of a fluorescently-labeled host cell membrane marker into the ApV and bacterial cholesterol content (18), which makes sense given that formation of cholesterol-rich ILVs in the ApV would be compromised in these cells. More experimentation is needed to determine if the proposed models for how A. phagocytophilum acquires cholesterol are mutually exclusive.

Cholesterol extraction from ILVs also depends on SM hydrolysis to ceramide. SM inhibits the NPC2-LBPA interaction that promotes NPC2-mediated cholesterol release from ILVs (24, 61). The negative charge of LBPA recruits hydrolases that are positively charged in an acidic environment, including acid sphingomyelinase (ASMase) (24). This enzyme hydrolyzes SM in ILVs to ceramide, which enables cholesterol secretion by NPC2 (24, 62). A. phagocytophilum promotes Golgi fragmentation to enhance delivery of TGN-derived SM-rich cargo into the ApV as ILVs. Similar to what was observed for Rab27b knockdown cells, disrupting traffic from the TGN impairs bacterial proliferation and infectious progeny production (17). Likewise, treatment with desipramine, a functional inhibitor of ASM, inhibits ApV maturation, bacterial proliferation, and infectious progeny production/release (15). By inducing Golgi fragmentation, A. phagocytophilum maximizes SM delivery to its MVB niche where ASM promotes SM hydrolysis to ceramide and consequent cholesterol liberation from ILVs for the bacterium to access for fueling its growth and the generation of infectious progeny.

Prior to this report, there was no understanding of how A. phagocytophilum exits host cells. By living in an MVB that acquires ALIX, ESCRT-III, Rab27a, and Munc13-4, it secures the exosome release pathway as a dissemination route. The acidic nature of the ApV potentially further benefits bacterial egress given that microenvironments having pHs in the range of the ApV increase exosome secretion (63). Consistent with MVB exosome secretion, Rab27a specifically associates with ApVs positioned at the plasma membrane and spilling bacteria into the extracellular milieu. Moreover, Nexinhib20 inhibits A. phagocytophilum DC exit targeting Rab27a-mediated release. Other intracellular pathogens have evolved to exploit MVB biogenesis machinery and might also coopt Rab27a. HIV and vaccinia virus budding from host cells requires ESCRT-III components (64, 65), while exosomal secretion of infectious prions is regulated by ESCRT-dependent and ESCRT-independent pathways (66). The apicomplexan parasite, Toxoplasma gondii, lives in a parasitophorous vacuole that acquires ESCRT machinery for transport of cholesterol and host proteins across its membrane (67–70). Ehrlichia chaffeensis, which is in the family Anaplasmataceae with A. phagocytophilum, similarly requires cholesterol and occupies a vacuole that receives cholesterol and host cell membranes, is full of ILVs, and is positioned adjacent to, but does not fuse with, MVBs (21, 71). Whether E. chaffeensis or T. gondii coopts exosome secretion for exit is unknown. The efficacy of Nexinhib20 in inhibiting A. phagocytophilum spread and the conserved reliance on MVB trafficking among diverse intracellular microbes make MVB biogenesis and exosome secretion attractive host-directed therapeutic targets for potentially treating a variety of infectious diseases.

In neutrophils, Rab27a helps control secretion of MVBs along with specific granules, secretory azurophilic granules, and tertiary granules (28). A. phagocytophilum infected neutrophils undergo persistent degranulation (i.e., exocytosis) (72), which, when considered with our data, suggests that the bacterium not only hitches a ride in MVBs but also stimulates exosome release to maximize its dissemination. A. phagocytophilum also likely exploits this pathway in its tick vector during transmission feeding. Extracellular vesicles released from Ixodes scapularis salivary glands exhibit protein profiles consistent with exosomes, modulate skin immunity at the feeding site, exhibit altered posttranslational modification profiles during A. phagocytophilum infection, and benefit A. phagocytophilum transmission (73). Overall, our findings, together with these reports, argue that the evolution of A. phagocytophilum as an obligate endosymbiont of ticks and animal reservoirs selected it for the ability to exploit the trans-phylum conserved MVB biogenesis-exosome release pathway throughout its enzootic cycle. Because this adaption contributes to its efficacy as a pathogen in humans and other accidental mammalian hosts, further investigation of the mechanism by which A. phagocytophilum exploits this pathway and whether it could be targeted *in vivo* by host-directed therapeutics are warranted.

## MATERIALS AND METHODS

### Cell lines and cultivation of A. phagocytophilum.

Uninfected and A. phagocytophilum NCH-1 strain infected human promyelocytic HL-60 cells (ATCC CCL-240; American Type Culture Collection [ATCC]) were cultured as previously described (30). Uninfected and infected RF/6A Macaca mulatta choroidal endothelial cells (CRL-1780; ATCC) were cultured in Dulbecco’s modified Eagle’s medium (DMEM) with l-glutamine, 4.5 g d-glucose and 100 mg sodium pyruvate (Gibco) supplemented with 10% (vol/vol) heat-inactivated fetal bovine serum (FBS) (Gemini Bioproducts), 1X minimal essential medium containing non-essential amino acids (Gibco) and 15 mM HEPES (Gibco) in a humidified incubator at 37°C with 5% atmospheric CO_2_.

### Human neutrophil isolation.

Isolation of human neutrophils from the peripheral blood of healthy donors was performed as previously described (15). All investigations using neutrophils obtained from human donor blood were conducted according to the principles expressed in the Helsinki Declaration. Informed consent was obtained from all subjects. Protocol HM11407 for obtaining donor blood for the purpose of isolating neutrophils has been reviewed and approved by the Virginia Commonwealth University Institutional Review Board with respect to scientific content and compliance with applicable research and human subject regulations.

### Infection assays.

HL-60 cells and human neutrophils were infected with A. phagocytophilum DC organisms that had been released from infected HL-60 cells by sonication and purified as described (30). To evaluate the ability of A. phagocytophilum to infect cells in the presence of Nexinhib20 (Sigma-Aldrich), HL-60 cells were treated with 1 μM Nexinhib20 or DMSO vehicle control in Iscove’s modified Dulbecco’s medium (Invitrogen) containing 10% (vol/vol) FBS for 1 h at 37°C, followed by incubation with DC organisms (53). Triplicate samples were analyzed by indirect immunofluorescence microscopy at 24 hpi to determine the number of ApVs per cell and the percentage of infected cells (53). The percentage of infected cells was normalized to that for vehicle-treated cells. To determine if Rab27a-mediated exocytosis is critical for A. phagocytophilum release, HL-60 cells were synchronously incubated with A. phagocytophilum DC organisms as previously described (7). Infected cells were seeded into 12-well plates and incubated at 37°C in a humidified incubator at 5% atmospheric CO_2_. At 24 hpi, aliquots from individual wells were collected and processed for immunofluorescence microscopy to determine the percentage of infected cells and the number of ApVs per cell. Each well was subsequently treated with 1 μM Nexinhib20 or DMSO for 6 additional hours. At 30 hpi, aliquots were collected and examined for infection. The remaining volume was collected and centrifuged for 5 min at 500 × *g*. Pellets were processed for qPCR. To assess for infectious progeny, the supernatant from each sample was retained and used to infect naive HL-60 cells as described (7). At 24 hpi, the recipient cells were processed for qPCR to quantify bacterial load. For GW4869 (Sigma-Aldrich) treatment, uninfected HL-60 cells were treated with 10 μM GW4869 or DMSO for 1 h before incubation with A. phagocytophilum DC organisms (7) in the continued presence of the GW4869 or DMSO. At 48 h, cells were harvested and processed for qPCR or immunofluorescence microscopy as described below. To assess the bacterial load as the mean fluorescent intensity of A. phagocytophilum P44 immunosignal, the fluorescence intensity was quantified for multiple fields of view in instances when P44 immunosignal overlapped with A. phagocytophilum inclusion DAPI signal. The number of cells per set area was approximately 600. The bacterial load was quantified by exporting the Leica Application Suite X (LAS X) data file to Microsoft Excel and averaging the intensity of P44 signal for each field of view per coverslip.

RF/6A cells were infected with A. phagocytophilum DC bacteria that had been naturally released from heavily (≥90%) infected RF/6A cells into the culture media and subsequently diluted in fresh media at a ratio of 1:5. Recipient RF/6A cells in 6-well plates were overlaid with 1 mL of the diluted A. phagocytophilum mixture, while RF/6A cells in 24-well plates received 300 μL. To maximize contact of DC organisms with the RF/6A cells, the plates were centrifuged at 1000 × *g* for 3 min. After 1 h incubation at 37°C in a humidified incubator with 5% atmospheric CO_2_, the cells were washed with 1X phosphate-buffered saline (PBS; 1.05 mM KH_2_PO_4_, 155 mM NaCl, 2.96 mM Na_2_HPO_4_ [pH 7.4]) to remove unbound bacteria followed by the addition of fresh media.

### Transfection.

pmCherry-Vimentin-N-18 was a gift from Dr. Michael Davidson (Addgene plasmid # 55158; http://n2t.net/addgene:55158; RRID:Addgene_55158). RF/6A cells grown to 70% to 90% confluence, were transfected with plasmid DNA using Lipofectamine-2000 or -3000 (Invitrogen) per the manufacturer’s instructions, and incubated at 37°C in a humidified incubator with 5% atmospheric CO_2_ for 24 to 48 h. Following transfection, RF/6A cells were synchronously infected with A. phagocytophilum for 24 h and processed for immunofluorescence microscopy.

### Western blot.

Western blotting of whole cell lysates was performed as previously described (74). Briefly, normalized amounts of eluates were resolved by SDS-PAGE in 4-to-20% mini-Protean gels (Bio-Rad) at 110 V for 15 min, followed by 200 V for 25 min. Following transfer onto nitrocellulose membranes, blots were blocked and probed with 5% (vol/vol) nonfat dry milk in Tris-buffered saline plus 0.05% Tween 20 (TBS-T). Primary antibodies used were rabbit anti-ALIX (Abcam [ab88388]; 1:1000), rabbit anti-Hgs (Abcam [ab155539]; 1:1000), rabbit anti-Rab11a (Abcam [ab88388]; 1:1000), rabbit anti-Rab27a (Abcam [ab55667]; 1:1000), rabbit anti-Rab27b (Cell Signaling Technology [ab44813]; 1:1000), mouse anti-GAPDH (Santa Cruz [sc365062]; 1:1000), mouse anti-β-actin (Santa Cruz [sc47778]; 1:1000), or antisera targeting A. phagocytophilum P44 or APH1235. Rabbit P44 antiserum was generated by New England Peptide. Rabbit anti-APH1235 (45) was a gift from Dr. Erol Fikrig of Yale University, New Haven, CT. Secondary antibodies were horseradish peroxidase-conjugated horse anti-mouse IgG or anti-rabbit IgG (Cell Signaling Technology; 1:10,000). All blots were incubated with chemiluminescent substrates, imaged, and processed as described (74).

### RT-qPCR and PCR.

Total cellular RNA was isolated using the RNeasy minikit (Qiagen). Sample RNA was eluted in 30 μL of RNase-free water and concentration and purity was confirmed using a spectrophotometer (NanoVue Plus, GE). Normalized RNA concentrations of up to 2 μg were DNase treated with amplification grade Dnase I (Invitrogen), following the manufacturer’s protocol. cDNA was synthesized following the iScript Reverse Transcription Supermix protocol (Bio-Rad). Genomic DNA depletion was confirmed using samples as template for PCR in the presence or absence of reverse transcriptase with primers specific for our housekeeping gene, *β-actin* (32) and MyTaq polymerase (Bioline). PCR amplified products were visualized by agarose gel electrophoresis. Using cDNA as template, qPCR was performed with SsoFast EvaGreen supermix (Bio-Rad) and primers specific for *β-actin* (32), *chmp4a* (5′-ACCAAGAATAAGAGAGCTGCCC-3′ and 5′-CAATGGCCTCACGCTGAAAC-3′), *chmp4b* (5′-CAAAAACAAGCGCGCAGCC-3′ and 5′-CGGTGTTGGTGTTGGCATTC-3′), *chmp4c* (5′-GCAACAGGATATCGCCC-3′ and 5′-TGCCATCAACTCATCCTCATCA-3′), and *rab39a* (5′-AGGAGCGGTTCAGATCAATAAC-3′ and 5′-AATCCGAAACGGCTGTACATA-3′). Thermal cycling conditions used were 95°C for 30 s followed by 39 cycles of 95°C for 5 s, and 57°C for 5 s. Amplification of *rab39a* required an annealing/extension temperature of 51°C. To determine the A. phagocytophilum DNA load, DNA was isolated from infected cells using the DNeasy blood and tissue kit (Qiagen). The bacterial DNA load was quantified using primers specific for A. phagocytophilum *16S rDNA* and *β-actin* (32), SsoFast EvaGreen, and 50 or 100 ng of template. Thermal cycling conditions used were 98°C for 2 min, followed by 39 cycles of 98°C for 5 s, and 55°C for 10 s. Relative expression or relative DNA load among samples was determined using the 2^−ΔΔCT^ method (75) as part of the CFX Maestro for Mac 1.0 software package (Bio-Rad).

### siRNA knockdown.

RF/6A cells grown to 70% to 90% confluence, were transfected with 30 pmol of ON-TARGET siRNA (Dharmacon) using Lipofectamine RNAiMAX (Invitrogen) per the manufacturer’s protocol. Custom designed siRNA oligonucleotides targeted *hgs* (5′-AGAGACAAGUGGAGGUAAAUU-3′ and 5′-UCAUGAAGGGUGGAGGGACAUU-3′), *alix* (5′-GAAGAAAUUUGGAGAAGAAUU-3′ and 5′-CCAAGAACUCUAAAGAAAUU-3′), *chmp4a* (5′-UGAAGAAGCAAUAACAGAAAUU-3′ and 5′-GGAUGAAGAUGAAGAAGCAUU-3′), *chmp4b* (5′-CGAUAAAGUUGAUGAGUUAUU-3′ and 5′-CGGAGGAGAUGUUAAGCAAAUU-3′), *chmp4c* (5′-GGGCAGAAGAAGAGGAUGAUU-3′ and 5′-UGGCAGAACUUGAAGAAUUUU-3′), *rab27a* (5′-AAACAUAAGCCAAGCAAUUUU-3′ and 5′-CCAAUAUACAGAUGGUAAAUU-3′), *rab27b* (5′-CAAAUGCU UAUUGUGAAAUU-3′ and 5′-AAAUGGAUCUUCAGGGAAAUU-3′), *rab39a* (5′-CUACAAAUGUUGAAGAAUCUU-3′ and 5′-GAAAGAUUGGCUAGAAGAAUU-3′), and *rab11a* (Dharmacon [ON-TARGETplus Pool Cat #L-004726-00-0005]). ON-TARGETplus non-targeting SMARTpool siRNA was used as a control (Dharmacon). RF/6A cells were synchronously infected 48 h posttransfection with A. phagocytophilum as described above. At designated time points, six-well plates were washed with 1× PBS and cells were collected using a cell scraper for isolating protein, DNA, or RNA. The percentage of infected cells, the number of ApVs per cell, *aph1235* expression, and infectious progeny were assessed as described above.

### Immunofluorescence and live cell microscopy.

RF/6A cells were seeded onto 12-mm glass coverslips (Electron Microscopy Sciences) in 24-well plates. At the appropriate postinfection time point, the cells were fixed in 4% (vol/vol) paraformaldehyde (Electron Microscopy Sciences) in PBS for 20 min followed by permeabilization in 0.5% (vol/vol) Triton X-100 (Fisher Scientific) in PBS. Samples were blocked in 5% (vol/vol) BSA (Fisher Scientific) in PBS for 1 h followed by probing with primary antibodies diluted in 1% (vol/vol) BSA in PBS. Primary antibodies used were mouse anti-LBPA clone 6C4 (MilliporeSigma [MABT837]; 1:250), rabbit anti-CHMP4B (Proteintech [13683-1-AP]; 1:200), mouse anti-CD63 (Abcam [ab59479]; 1:200), rabbit anti-ALIX (1:200), rabbit anti-Hgs (1:200), mouse anti-Rab27a (1:200), rabbit anti-Rab27b (1:200), rabbit anti-UNC13D (Munc13-4) (Abnova [H00201294-D01P]; 1:200), rabbit anti-P44, mouse P44 monoclonal antibody 20B4 (a gift from Dr. J. Stephen Dumler of Uniformed Services University for the Health Sciences) (76), rat anti-APH1235, and rat anti-APH0032. Rat anti-APH1235 and anti-APH0032 were generated by immunizing Sprague-Dawley rats with His-tagged APH1235 and APH0032 following previously described protocols (74). Secondary antibodies conjugated to Alexa Fluor fluorochromes were obtained from Invitrogen. DAPI (4′,6′-diamidino-2-phenylindole, Invitrogen) was used per the manufacturer’s instructions to stain host cell nuclei and A. phagocytophilum nucleoids. Coverslips were mounted using Prolong Gold Anti-fade reagent (Invitrogen) and imaged at room temperature. Images were obtained using a Zeiss LSM 700 laser scanning confocal microscope (Zeiss).

For live cell imaging to assess the relative pH of the ApV, RF/6A cells were cultured in 24-well glass bottom plates (Greiner Bio-One Monroe) and transfected with plasmid encoding mCherry-Vimentin-N-18 as described above. At 24 h posttransfection, the cells were infected with A. phagocytophilum, and pH was analyzed 24 h later using LysoSensor Green DND-189 and BCECF (Molecular Probes Invitrogen Detection Technologies), according to the manufacturer’s instructions. Briefly, Hoechst 33342 (Thermo Fisher Scientific) was incubated with RF/6A cells for 10 min at 37°C prior to incubation with either time-sensitive pH indicator. The cells were washed with 1× PBS and subsequently treated with LysoSensor Green DND-189 or BCECF mixed with medium for 3 or 15 min, respectively, at 37°C. The cells were washed, replenished with fresh medium, and immediately imaged at room temperature with a TCS SP8 microscope (Leica Microsystems) affixed with an Andor iXon Life 888 EMCCD camera (Oxford Instruments) and a 63× water-immersion objective with a 1.2 numeric aperture.

LSCM and live cell image processing was accomplished using LAS X (Leica Microsystems) software (version 3.7.4.23463) and/or ImageJ (NIH). Line-profile analyses were performed on single Z-sections using ImageJ and relative signal intensity profiles were generated for each channel by normalizing images to 0.1% saturation. The output of the fluorescent intensity values for each channel along the line-profile were normalized to the highest intensity value. To assess three-dimensional depth of Rab27a-positive ApVs, a Z-projection was generated as an array of 16 successive Z-plane images from bottom to top obtained using the LSM 700 laser scanning confocal microscope. Temporal color code was applied to the Z-projection using the fire look up table in Fiji (https://fiji.sc/) to visualize the three-dimensional depth of Rab27a, vimentin, the host cell nucleus, and bacterial nucleoid signals.

### Transmission electron microscopy.

Approximately 0.2 x 10^6^ RF/6A cells in a volume of 2 mL were seeded into 60 × 15 mm Permanox dishes (Electron Microscopy Sciences), followed by infection with A. phagocytophilum DC organisms. At 24 h, the cells were washed with 1 × PBS and fixed in 2.5% (vol/vol) glutaraldehyde in 0.1M sodium cacodylate buffer at room temperature. Next, samples were dehydrated in by successive incubations in 50%, 70%, 80%, 95%, and 100% acetone. Each incubation was performed three times for 10 to 15 min. Following dehydration, samples were infiltrated with a 50:50 mix of acetone and PolyBed 812 resin (Polysciences, Inc.) overnight. The next day, samples were infiltrated further with pure PolyBed 812 resin for 8 h followed by a second incubation in PolyBed 812 resin overnight. Finally, samples were embedded in Permanox dishes and cured at 55 to 60°C for 2 days. Sectioning was accomplished using a Leica EM UC6i Ultramicrotome. The resulting 90-nm-thick sections were placed on copper grids and stained with 5% uranyl acetate and Reynold’s lead citrate. Grids were visualized using a JEOL JEM-14000 TEM (JEOL USA, Inc.) with the Gatan OneView digital camera (Gatan Inc). In some experiments, a 6-nm BSA-gold tracer (Electron Microscopy Sciences) was incubated with the infected cells at 20 h for 4 h at 37°C. The cells were washed three times with 1% BSA in 1 × PBS to remove unbound BSA-gold. Fresh media was added to cells for an additional 30 min to allow the tracer to internalize. A final 1 × PBS wash was conducted, after which samples were fixed in 1% (vol/vol) osmium tetroxide in 0.1 M cacodylate buffer for 1 h and subjected to three 10-min rinses in 0.1 M cacodylate buffer. The samples were then processed further for viewing by transmission electron microscopy.

### Statistical analysis.

The Student's *t* test (paired), performed using the Prism 8.0 (GraphPad), was used to assess statistical significance between pairs. *P* values of < 0.05 were considered statistically significant.
