# Supplementary figures and images for: The Obligate Intracellular Bacterial Pathogen Anaplasma phagocytophilum Exploits Host Cell Multivesicular Body Biogenesis for Proliferation and Dissemination
Source: mBio. 2022 Nov 21;13(6):e02961-22. doi: 10.1128/mbio.02961-22 (PMC9765717; doi:10.1128/mbio.02961-22)

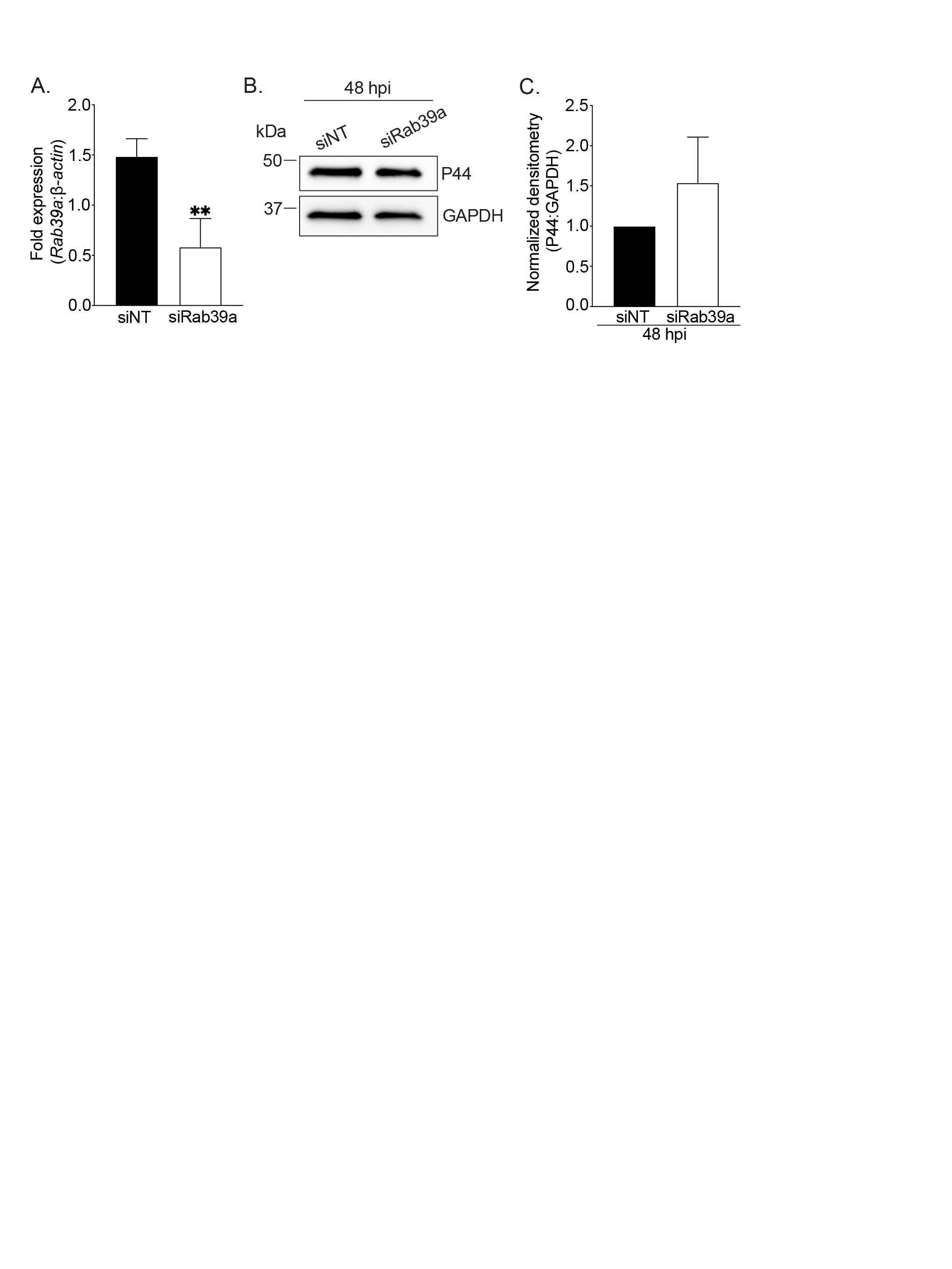

Supplement: FIG S1 [file mbio.02961-22-s0001.tif]

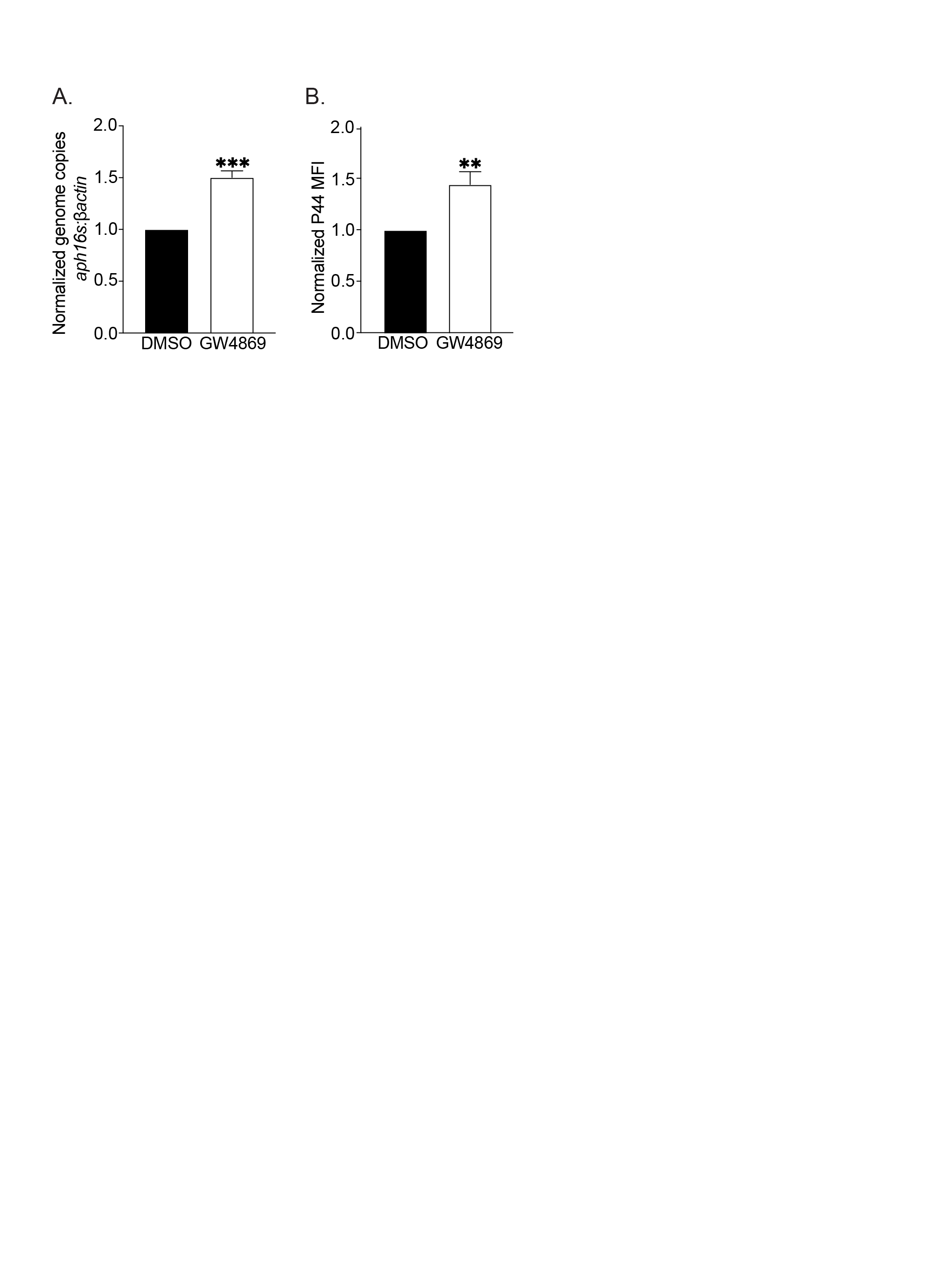

Supplement: FIG S2 [file mbio.02961-22-s0002.tif]

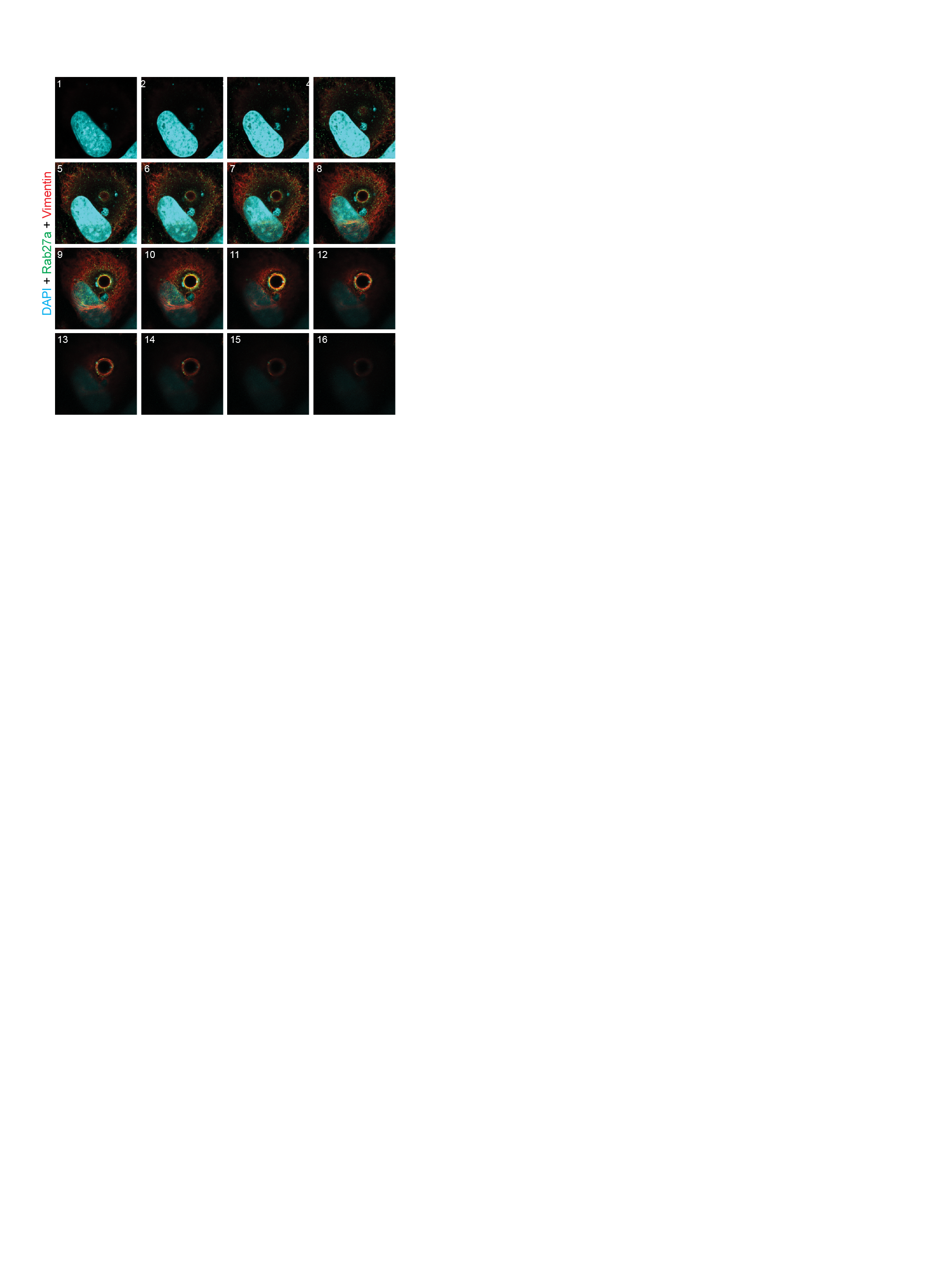

Supplement: FIG S3 [file mbio.02961-22-s0003.tif]

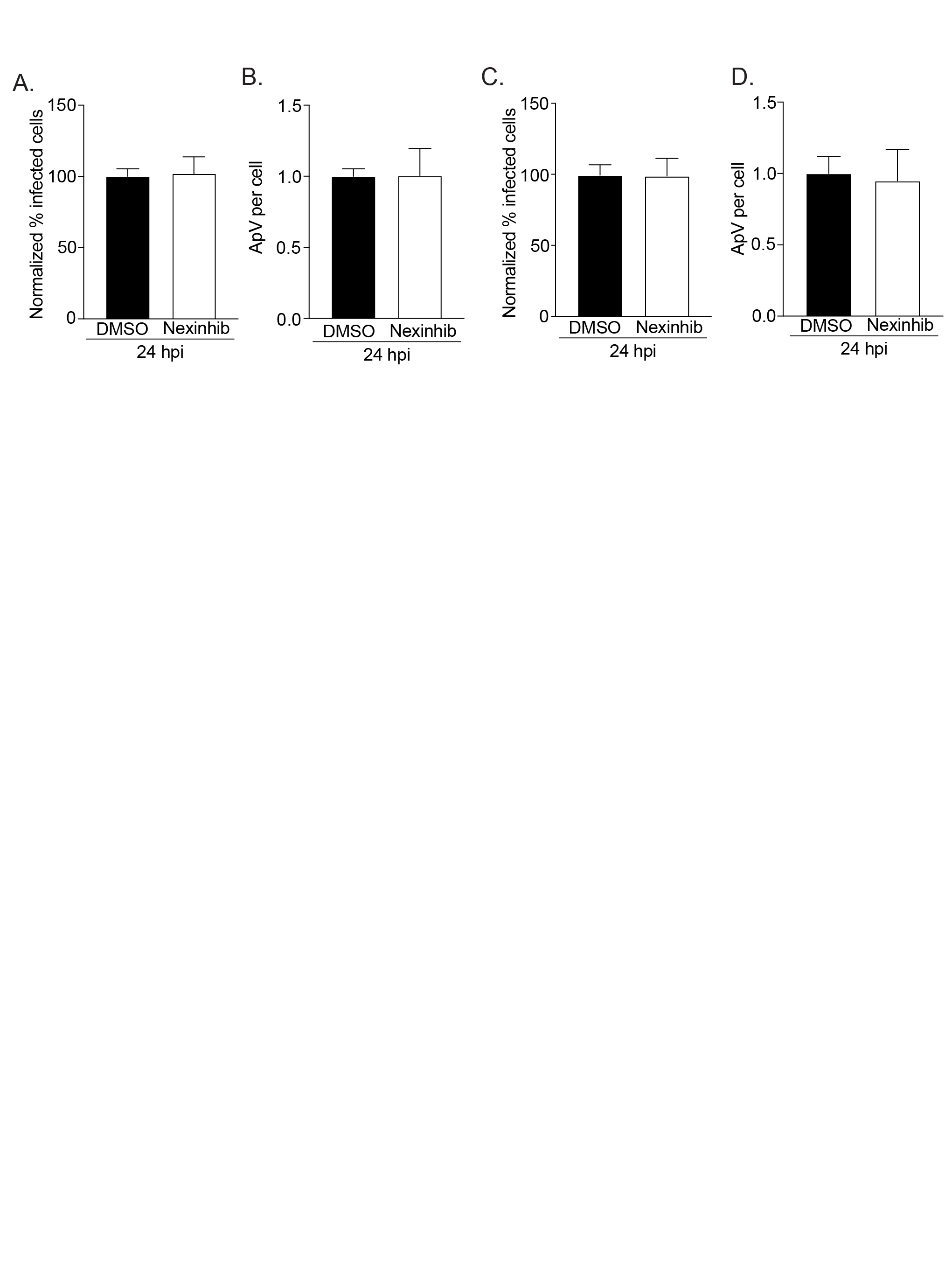

Supplement: FIG S4 [file mbio.02961-22-s0004.tif]

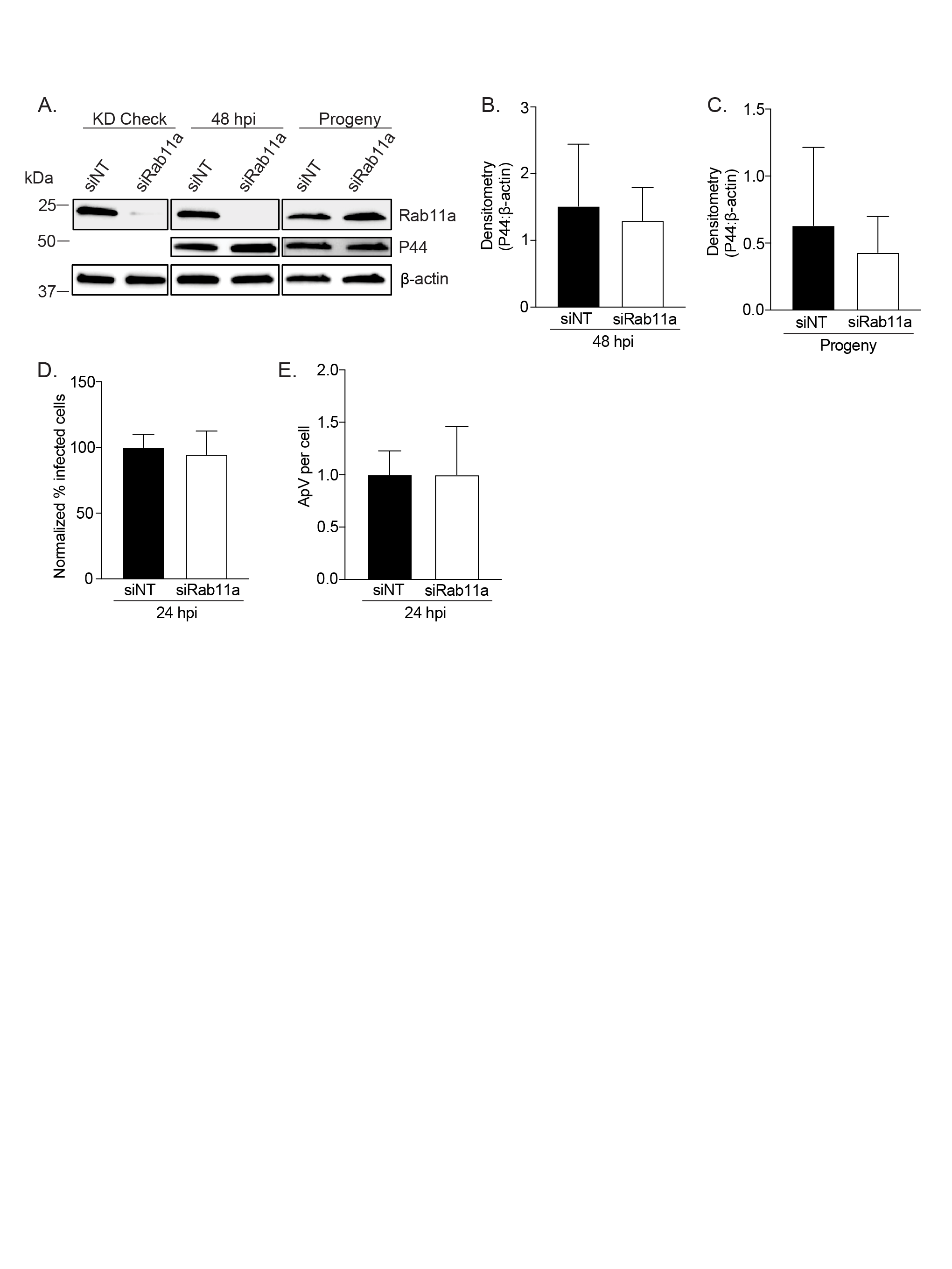

Supplement: FIG S5 [file mbio.02961-22-s0005.tif]
